# Explaining trends in coronary heart disease mortality in different socioeconomic groups in Denmark 1991-2007 using the IMPACT_SEC_ model

**DOI:** 10.1371/journal.pone.0194793

**Published:** 2018-04-19

**Authors:** Albert Marni Joensen, Torben Joergensen, Søren Lundbye-Christensen, Martin Berg Johansen, Maria Guzman-Castillo, Piotr Bandosz, Jesper Hallas, Eva Irene Bossano Prescott, Simon Capewell, Martin O'Flaherty

**Affiliations:** 1 Aalborg University Hospital, Department of Cardiology, Aalborg, Denmark; 2 Research Center for Prevention and Health, Centre of Health, The Capital Region, Copenhagen, Denmark; 3 Department of Public Health, Faculty of Health and Medical Sciences, University of Copenhagen, Copenhagen Denmark; 4 Faculty of Medicine, Aalborg University, Aalborg, Denmark; 5 Unit of Clinical Biostatistics, Aalborg University Hospital, Aalborg, Denmark; 6 University of Liverpool, Department of Public Health and Policy, Liverpool, United Kingdom; 7 Department of Preventive Medicine and Education, Medical University of Gdańsk, Gdańsk, Poland; 8 Clinical Pharmacology, Department of Public Health, University of Southern Denmark, Odense, Denmark; 9 Bispebjerg University Hospital, Capital Region of Denmark, Copenhagen, Denmark; CUNY, UNITED STATES

## Abstract

**Aim:**

To quantify the contribution of changes in different risk factors population levels and treatment uptake on the decline in CHD mortality in Denmark from 1991 to 2007 in different socioeconomic groups.

**Design:**

We used IMPACT_SEC_, a previously validated policy model using data from different population registries.

**Participants:**

All adults aged 25–84 years living in Denmark in 1991 and 2007.

**Main outcome measure:**

Deaths prevented or postponed (DPP).

**Results:**

There were approximately 11,000 fewer CHD deaths in Denmark in 2007 than would be expected if the 1991 mortality rates had persisted. Higher mortality rates were observed in the lowest socioeconomic quintile. The highest absolute reduction in CHD mortality was seen in this group but the highest relative reduction was in the most affluent socioeconomic quintile. Overall, the IMPACT_SEC_ model explained nearly two thirds of the decline in. Improved treatments accounted for approximately 25% with the least relative mortality reduction in the most deprived quintile. Risk factor improvements accounted for approximately 40% of the mortality decrease with similar gains across all socio-economic groups. The 36% gap in explaining all DPPs may reflect inaccurate data or risk factors not quantified in the current model.

**Conclusions:**

According to the IMPACT_SEC_ model, the largest contribution to the CHD mortality decline in Denmark from 1991 to 2007 was from improvements in risk factors, with similar gains across all socio-economic groups. However, we found a clear socioeconomic trend for the treatment contribution favouring the most affluent groups.

## Introduction

Coronary heart disease (CHD) mortality has declined substantially during recent decades in Denmark and other Western countries [1]. CHD, however, remains one of the leading causes of death, morbidity and economic burden for the Danish health care systems and in the world [2,3]. This decline has been attributable to decreases in prevalence of risk factors for CHD and to improvements of medical treatment and treatment uptake [4–8]. Some studies have reported that population-level changes to risk factors such as smoking, cholesterol, hypertension, physical inactivity, obesity and diabetes mellitus are responsible for 45% to 75% of the decline in CHD mortality, while treatments improvement could be responsible for 25% to 50% of the decline [4–6].

Previous studies have demonstrated that the decrease in CHD mortality has not been equally beneficial for all socioeconomic groups [9,10]. The most deprived groups have the highest reductions in CHD mortality in absolute numbers whereas the most affluent group benefit from the highest reductions in relative terms. This has led to an overall lower CHD mortality but a relative bigger gap between the affluent and deprived groups. Studies from UK have shown that inequality in health also has significant economic costs due to loss of production, loss of tax payment and higher expenses to welfare and health care (https://heartuk.org.uk/files/uploads/Bridging_the_Gaps_Tackling_inequalities_in_cardiovascular_disease.pdf).

Denmark, considered as one of the most socioeconomic equal societies in the western world with the lowest Gini coefficient in the study period (http://dx.doi.org/10.1787/420515624534 and http://dx.doi.org/10.1787/422066332325), has a taxpayer funded health care system given free access to most of the health care services. Despite of this it has been shown that there are socioeconomic differences in the state of health in Denmark and in uptake of different medical treatments [11,12].

In this study, we aim to quantify the contribution of changes in different population risk factors and treatment uptake on CHD mortality in Denmark from 1991 to 2007 in different socioeconomic groups.

## Methods

The IMPACT model is a deterministic, cell-based model that has been developed to help explain the contribution of the change in different risk factors and treatment uptakes on CHD mortality [4,6]. The model uses information from many different data sources on patient numbers, uptake of evidence based medical treatments and the case fatality reduction for each treatment. Also, the model includes prevalence of risk factors from two time points in the current study, 1991 and 2007, and uses mortality risk from each risk factor. Thus, the model calculates how much changes in treatments and risk factor prevalence has contributed to the change in CHD mortality in the study period. Recently the IMPACTsec model has been developed to also quantify the impact of socioeconomic inequalities in risk factor prevalence and treatment uptake [9,10] on CHD mortality.

The extended IMPACTsec model also included all the major risk factors for CHD: smoking, systolic blood pressure, total cholesterol, body mass index (BMI), diabetes, physical inactivity; plus medical and surgical treatments currently in use in nine patient groups [9,10].

### Population

The Danish IMPACTsec model included the Danish population between 25–84 years in 1991 and 2007. The population was divided into 60 different groups based on gender, six different age-groups (25–34, 35–44, 45–54, 55–64, 65–74 and 75–84), and five socioeconomic-level groups based on quintiles of personal financial income.

Information of population counts was obtained from the Danish Central Office of Civil Registration which contains complete information on vital status, date of birth and a unique personal 10-digit code that makes it possible to merge information from different registers on an individual level [13]. Financial income data was obtained from Statistic Denmark. Based on gender and age specific personal tax-income information, the population was divided into socioeconomic quintiles (secq groups) ranged from the most affluent to the most deprived.

### Deaths prevented or postponed

The numbers of CHD deaths expected in 2007 were calculated by multiplying the age and sex-specific CHD mortality in 1991 by the relevant population counts for 2007. Summing over all strata then yielded the expected number of deaths in 2007 had mortality rates remained unchanged.

The difference between the number of expected and observed deaths then represented the decline in mortality or the total number of CHD deaths prevented or postponed to be explained (DPPs) by the combined changes in risk factor levels and treatment uptake between 1991 and 2007.

Data on CHD deaths (1991: International Classification of Diseases (ICD)-8 code 410–414 and 427; 2007: ICD-10 code I20-25 and I50) were obtained from The Danish Cause of Death Registry

### Disease groups and treatments uptake

The Danish IMPACTsec model includes nine mutually exclusive disease groups: 1) ST elevation acute myocardial infarction (STEMI), 2) Non-ST elevation acute myocardial infarction (NSTEMI), 3) previous myocardial infarction eligible for secondary prevention, 4) previous coronary artery bypass grafting surgery (CABG) or Percutaneous Coronary Intervention (PCI) eligible for secondary prevention, 5) stable angina pectoris, 6) hospital admission for congestive heart failure (CHF), 7) CHF treated in the community, 8) patients eligible for primary prevention of hypercholesterolemia, and 9) patients eligible for primary prevention of hypertension.

When individuals had more than one cardiac diagnosis, they were categorized into only one disease-group representing the highest mortality risk.

For each disease group we specified relevant treatments. To quantify mortality risk reduction due to treatment we used the number of eligible patients for each of the nine mutually exclusive disease-groups, the change in treatment uptake rate for relevant treatments in the two index years and the case fatality reduction for each treatment.

Example: Net effects for treatments

Calculating net effects for aspirin use in STEMI cases in men aged 55–64 in the most deprived quintile

With an estimated total of 147 men aged 55–64 with STEMI in the most deprived quintile, uptake rate in 2007, 97% and uptake rate in 1991, 14%, a relative risk reduction of 23%, a one-year case fatality rate of 34%, and 100% compliance, the total number of DPPs for aspirin was:

Patient numbers × (treatment uptake_2007_—treatment uptake_1991_) × compliance × relative mortality reduction × one year case fatality:

147x(97%−14%)x1x23%x34%=9.5DPPs

This calculation was repeated for each age-gender-secq group and we incorporated a Mant and Hicks adjustment for multiple medications within each patient group. (For a detailed overview for treatments for each disease group and the Mant and Hicks adjustment, (please see S1 Technical Appendix).

Information of hospital admissions for CHD diagnoses (STEMI, NSTEMI and CHF), was obtained from The Danish National Patient Registry. Number of patients in the community with CHF and angina pectoris and patients eligible for secondary prevention therapy were also obtained from Danish National Patient Registry and was defined as persons with a hospital admission for the relevant diagnosis within the previous 11 years.

Number of patients eligible for primary therapy (hypercholesterolemia and hypertension) was based on information from cohort studies Copenhagen City Heart Studies (CCHS) 3 and 4 [14] and was defined as the proportion of persons with either self-reported hypercholesterolemia/hypertension and/or measurement of a serum-total cholesterol > 5 mmol/l or a systolic blood pressure > 140 mmHg.

Treatment uptake of pharmacological treatment in 2007 was obtained from The Danish Medical Agency Registry. Since there was no national prescription registry in Denmark in 1991 we used the regional prescription registry from Funen [15] to estimate treatment uptake rates for 1991 and assumed that this was representative for the whole country. There is no Danish registry with data on uptake of in-hospital pharmacological treatment so we performed a survey among patients discharged with diagnoses of acute coronary syndrome (ACS) and CHF. Information on invasive treatment with CABG and PCI was obtained from the National Patient Registry. One year case fatality rates of the diseases and relative risk reduction for each treatment was obtained from previous observational and randomized studies similar with other IMPACTsec models [9,10].

### Risk factors

In the Danish IMPACTsec model, we included six different major cardiovascular risk factors. For risk factors measured in a continuous scale (serum cholesterol, blood pressure and BMI), we used a regression based approach using independent age-sex specific coefficients for mortality benefit for each unit of change in the risk factors from the base year to the final year.

Example: Estimation of DPPs from risk factor changes using regression method

Among women aged 55–64 in the most deprived quintile the number of expected death was 176 in 2007. Mean total cholesterol in this group fell by an estimated 1.28 mmol/l (from 7.04 in 1991 to 5.76 in 2007). The largest meta-analysis reports an estimated age-sex specific reduction in mortality of 35% for every 1 mmol/l reduction in total cholesterol [16], generating a logarithmic coefficient of -0.431 (i.e. natural logarithm of (1–0.35)). The subsequent reduction in CHD deaths due to cholesterol reduction between 1991 and 2007 was then estimated as:

$$\begin{array}{l} \left(1-\left(exponential\;(regression\;coefficient\times absolute\;change)\right)\right)\times expected\;deaths\;in\;2007:\\ \left(1-\left(exponential\;\left(-0.431\times 1.28\right)\right)\right)\times 176=75\;DPPs \end{array}$$

This calculation was then repeated for each age-gender-secq group.

For dichotomous risk factors (smoking, physical inactivity and diagnosed diabetes mellitus) we used a population attributable risk fraction (PARF) approach using independent age-sex specific estimates for relative risks. PARF, which can be interpreted as the proportion by which the mortality rate from CHD would be reduced if the exposure was eliminated [17], was calculated as:

PARF=[P×(RR−1)]/[1+P×(RR−1)]

Where P is the prevalence of the risk factor and RR is the relative risk for CHD mortality associated with risk factor presence.

Example: Estimating DPPs from risk factor change–PARF approach for binary risk factors

The prevalence of diabetes among men in the most deprived quintile aged 65–74 years was 4.3% in 1991 and 8.2% in 2007. Assuming a relative risk of 1.86, the PARF at the national level for men aged 65–74 was 0.036 in 1991 and 0.066 in 2007.

The DPPs was therefore:

$$\begin{array}{l} expected\;CHD\;deaths\;in\;2007\times \left(PARF_{1991}–PARF_{2007}\right):\\ 874\times \left(0.036–0.066\right)=-26\;DPPs \end{array}$$

A negative sign for the DPPs denotes deaths brought-forward due to the increase in diabetes prevalence. The calculation was then repeated for each age-gender-secq group.

For calculation of the joint mortality benefits from risk factors, we used a cumulative (rather than an additive) risk reduction approach (see S1 Technical Appendix).

Information on risk factors was achieved through population cohort studies CCHS 3 and 4 described in details elsewhere [14]. Briefly, the cohort study invited over 20.000 men and women randomly drawn from Copenhagen Population Register in 1976 to participate with the purpose to describe the distribution of cardiovascular risk factors in the population and to examine these risk factors relation to morbidity and mortality. At the following examination in 1981–83, 1991–94 (CCHS 3) and 2002–3 (CCHS 4) the study population was supplemented with the youngest age groups while all previous participants alive and living in Denmark were invited. All subjects filled in a self-administered questionnaire (concerning e.g. smoking habits and physical inactivity), had a physical examination (BMI, blood pressure), and had a non-fasting venous blood sample (lipids). As we only had risk factors measurements for 1993 and 2003 we calculated gradients to estimate values for 1991 and 2007. Data on prevalence of DM was obtained from The Danish National Patient Registry.

### Sensitivity analysis

We calculated 95% empirical intervals around the model output (that is, deaths prevented or postponed) by using Monte Carlo simulation, as in health economic evaluation studies [18]. This calculation involved replacing all fixed input parameters used in the model by appropriate probability distributions and repeatedly recalculating the model output with values sampled from the defined input distributions. We used the Excel add-in Ersatz software (www.epigear.com) to do 10,000 runs to determine the 95% uncertainty intervals of the deaths prevented or postponed (2.5th and 97.5th centile values corresponding to the lower and upper limits) [10].

To control for possible selection bias regarding data collection from the cohort study with repeated examinations, for sensitivity analyses we also substituted estimates for risk factors fraction obtained from the CCHS cohort studies with estimates from cross sectional population surveys Dan-MONICA III (1991) and Health 2006 (2006–7) [19].

DPPs explained by the model could be positive (i.e., deaths averted) or negative (i.e., additional deaths in 2007 relative to 1991). Any differences between the DPPs explained by the model and the total DPPs for each secq group were assumed to reflect either imprecision in our model parameters or omission of other, unmeasured risk factors [9,10].

More details on the IMPACTsec model methodology, including the data sources for this study are described in details in S1 Technical Appendix.

## Results

**Fig 1:**
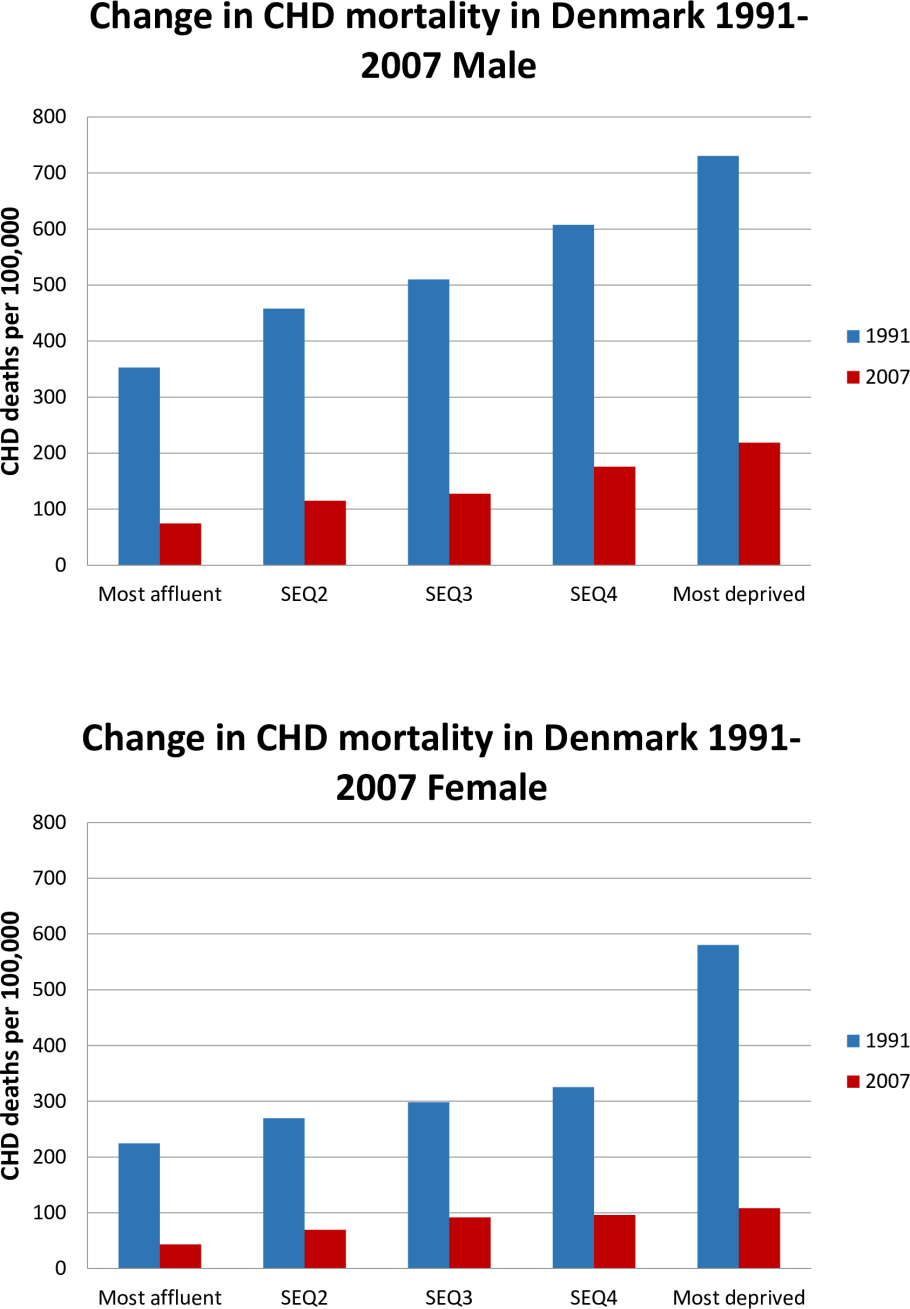
Change in CHD mortality in Denmark 1991–2007 for males and female.

As expected, CHD mortality rates were higher in males than females (Table 1). From 1991 to 2007 we observed a decline of nearly 75% in CHD mortality (indirect age standardization) for the age group 25–84 years in Denmark for both men and women. A decline was seen for all socioeconomic groups but differed somewhat in magnitude across gender-age-secq groups. As shown in Fig 1, the largest absolute reduction in mortality was among the most deprived women where the death rates were extremely high in 1991 compared to the other quintiles. The relative CHD mortality reductions ranged from nearly 80% to below 70% and, with the notable exception of the most deprived women, was overall higher in the more affluent for both females and males (Table 1).

**Table 1:** Coronary heart disease mortality rates in Denmark in 1991 and 2007, for the whole nation and divided into socio-economic quintiles. Also calculated numbers of deaths prevented or postponed (DPPs) in 2007 if the death-rates had been the same in 2007 as in 1991.

	Year	Denmark		Most Affluent*	secq2*	secq3*	secq4*	Most Deprived*
**Male**								
**Population (000s)**	**1991**		1555	311	311	311	311	311
	**2007**		1700	340	340	340	340	340
**Observed CHD deaths**	**1991**		8272	1098	1425	1587	1890	2273
	**2007**		2421	253	392	434	598	745
**Age-standardised rate (per 100,000)**	**1991**		532	353	458	510	608	730
	**2007**		138	73	114	126	170	208
**Expected deaths**^**†**^	**2007**		9396	1239	1610	1801	2166	2580
**DPPs**^**‡**^	**1991–2007**		6975	986	1219	1367	1568	1836
**Absolute mortality rate reduction (per 100,000)**	**1991–2007**		394	280	344	384	438	522
**Relative mortality rate reduction (%)**	**1991–2007**		74.2	79.6	75.7	75.9	72.4	71.1
**Female**								
**Population (000s)**	**1991**		1647	329	329	329	329	329
	**2007**		1760	352	352	352	352	352
**Observed CHD deaths**	**1991**		5597	741	889	983	1072	1913
	**2007**		1462	158	253	330	340	381
**Age-standardised rate (per 100,000)**	**1991**		340	225	270	298	325	581
	**2007**		89	48	77	100	103	116
**Expected deaths**^**†**^	**2007**		5575	728	882	992	1083	1890
**DPPs**^**‡**^	**1991–2007**		4135	575	638	669	744	1509
**Absolute mortality rate reduction (per 100,000)**	**1991–2007**		251	177	193	198	222	465
**Relative mortality rate reduction (%)**	**1991–2007**		74.1	79.0	72.3	67.5	68.7	79.8
**Total**								
**Population (000s)**	**1991**		3202	641	641	641	641	641
	**2007**		3461	692	692	692	692	692
**Observed CHD deaths**	**1991**		13879	1840	2316	2572	2964	4187
	**2007**		3870	408	638	759	939	1128
**Age-standardised rate (100,000)**	**1991**		433	287	361	402	463	654
	**2007**		112	60	95	112	136	161
**Expected deaths**^**†**^	**2007**		14981	1969	2494	2794	3251	4473
**DPPs**^**‡**^	**1991–2007**		11111	1561	1857	2036	2313	3345
**Absolute mortality rate reduction (per 100,000**	**1991–2007**		321	227	266	290	327	493
**Relative mortality rate reduction (%)**	**1991–2007**		74.2	79.3	74.4	72.9	71.1	74.8

* Socioeconomic quintiles based on financial income, with the most affluent group number 1 and the most deprived number 5.

^†^ Expected deaths: CHD deaths expected in 2007 had 1991 CHD rates remained.

^‡^ DPPs, deaths prevented or postponed. DPPs: expected–observed deaths in 2007.

The Danish IMPACTsec model could explain almost two third (95% CI 59.7–68.6%) of all DPPs. Thus, 23.8% (95% CI 20.9–27.5%) of the 11,111 DPPs could be explained by higher treatment uptake whereas the contribution of risk factors could explain 40.1% (95% CI 37.1–42.9%, Tables 2 and 3). The best model fit was in the middle sec quintile explaining 77% of all DPPs whereas it could only explain 53% and 61% in the most deprived and affluent sec quintile, respectively, (please see S1 Technical Appendix). The largest contribution of treatment was found within in-hospital CHF (4.4%) and CHF in the community (6.2%) (Table 2). Among the risk factors the largest contribution was from reduction in prevalence of hypercholesterolemia (24.1%) and smoking (9.9%). Lower systolic blood pressure and less physical inactivity contributed with 5.2% and 3.9%, respectively. Increase in BMI and prevalence of DM resulted in an increase of CHD mortality of 1.0% and 2.1%, respectively (Table 3).

**Table 2:** Number of coronary heart disease deaths prevented or postponed from 1991 to 2007 due to changes in treatment uptake in different patient groups in Denmark, *stratified by socioeconomic quintiles*.

Patient Groups	Deaths Prevented or Postponed							
	*N*	Percent^a^ Lower limit	Percent^a^ Upper limit	*n* Most Affluent	*n* secq2	*n* secq3	*n* secq4	*n* Most Deprived
**STEMI**	**127**	***73***	***233***	**19**	**24**	**25**	**30**	**29**
(%)^b^	**(1.1)**	*(0*.*7)*	*(2*.*1)*	(1.2)	(1.3)	(1.2)	(1.3)	(0.9)
**NSTEMI**	**181**	***98***	***347***	**32**	**36**	**39**	**39**	**42**
(%)^b^	**(1.6)**	*(0*.*9)*	*(3*.*1)*	(2.1)	(1.9)	(1.9)	(1.7)	(1.2)
**Secondary prevention post MI**	**412**	***329***	***519***	**62**	**78**	**85**	**93**	**93**
(%)^b^	**(3.7)**	*(3*.*0)*	*(4*.*7)*	(4.0)	(4.2)	(4.2)	(4.0)	(2.8)
**Secondary prevention post revascularisation**	**192**	***149***	***247***	**31**	**36**	**41**	**43**	**42**
(%)^b^	**(1.7)**	*(1*.*3)*	*(2*.*2)*	(2.0)	(1.9)	(2.0)	(1.9)	(1.3)
**Chronic stable angina**	**242**	***193***	***306***	**36**	**47**	**51**	**55**	**54**
(%)^b^	**(2.2)**	*(1*.*7)*	*(2*.*8)*	(2.3)	(2.5)	(2.5)	(2.4)	(1.6)
**Heart failure–hospital**	**420**	***357***	***506***	**46**	**68**	**87**	**102**	**117**
(%)^b^	**(3.8)**	*(3*.*2)*	*(4*.*6)*	(3.0)	(3.6)	(4.3)	(4.4)	(3.5)
**Heart failure–community**	**629**	***533***	***737***	**85**	**109**	**128**	**148**	**159**
(%)^b^	**(5.7)**	***(4*.*8)***	***(6*.*6)***	(5.4)	(5.9)	(6.3)	(6.4)	(4.8)
**Hypertension treatment**	**114**	***60***	***198***	**21**	**21**	**22**	**25**	**24**
(%)^b^	**(1,0)**	*(0*.*5)*	*(1*.*8)*	(1.3)	(1.1)	(1.1)	(1.1)	(0.7)
**Hyperlipidemia treatment (statins)**	**326**	***168***	***593***	**63**	**65**	**66**	**68**	**63**
(%)^b^	**(2.9)**	*(1*.*5)*	*(5*.*3)*	(4.0)	(3.5)	(3.3)	(3.0)	(1.9)
**A: Total treatment contribution**	**2,642**	***2*,*323***	***3*,*058***	**395**	**484**	**545**	**604**	**622**
(%)^b^	(23.8)	*(20*.*9)*	*(27*.*5)*	(25.3)	(26.1)	(26.8)	(26.1)	(18.6)

^a^ 95% uncertainty interval corresponds to the lower (2.5th percentile) and upper (97.5th percentile) limits of the uncertainty analysis. These are shown in italics to indicate range around the central estimate of percent of DPPs explained.

^b^ Numbers in brackets are the relative contribution of total DPPs for the treatment of the respective disease-group.

**Abbreviations:** MI, myocardial infarction; NSTEMI, non-ST-segment elevated myocardial infarction; secq, socio-economic quintiles; STEMI, ST-segment elevated myocardial infarction.

**Table 3:** Number of coronary heart disease deaths prevented or postponed from 1991 to 2007 due to changes in risk factor prevalence in Denmark, *stratified by deprivation quintiles*.

Risk Factors	Deaths prevented or postponed				
	*N*	Percent Lower Limit^b^	Percent Upper Limit^b^	*n* Most Affluent	*n* secq2	*n* secq3	*n* secq4	*n* Most Deprived
**Current smoking**	**1,105**	*919*	*1288*	**146**	**249**	**226**	**220**	**264**
(%)^a^	(9.9)	*(8*.*3)*	*(11*.*6)*	(9.3)	(13.4)	(11.1)	(9.5)	(7.9)
**Physical inactivity**	**437**	*331*	*558*	**35**	**54**	**61**	**133**	**154**
(%)^a^	(3.9)	*(3*.*0)*	*5*.*0*	(2.2)	(2.9)	(3.0)	(5.7)	(4.6)
**Systolic blood pressure, mmHg**^**c**^	**581**	*397*	*755*	**51**	**45**	**346**	**159**	**−20**
(%)^a^	(5.2)	*(3*.*6)*	*(6*.*8)*	(3.3)	(2.4)	(17.0)	(6.9)	(-0.6)
**Total cholesterol, mmol/l**^**d**^	**2,679**	*2*,*531*	*2*,*815*	**348**	**435**	**479**	**561**	**856**
(%)^a^	(24.1)	*(22*.*8)*	*(25*.*3)*	(22.3)	(23.4)	(23.5)	(24.3)	(25.6)
**Body mass index**	**−113**	*-169*	*-55*	**−6**	**−26**	**−35**	**−24**	**−23**
(%)^a^	(−1.0)	*(-1*.*5)*	*(-0*.*5)*	(-0.4)	(-1.3)	(-1.7)	(-1.0)	(-0.7)
**Diabetes mellitus**	**−231**	*-274*	*-188*	**−13**	**−25**	**−46**	**−65**	**−82**
(%)^a^	(−2.1)	*(-2*.*5)*	*(-1*.*7)*	(-0.8)	(-1.4)	(-2.3)	(-2.8)	(-2.4)
**Total risk factors contribution**	**4,453**	*4*,*119*	*4*,*766*	**561**	**731**	**1,032**	**986**	**1,150**
(%)^a^	(40.1)	*(37*.*1)*	*(42*.*9)*	36.0	39.4	50.7	42.6	34.4

^a^ Numbers in brackets are the relative contribution of total DPPs for the treatment of the respective disease-group.

^b^ 95% uncertainty interval corresponds to the lower (2.5th percentile) and upper (97.5th percentile) limits of the uncertainty analysis.

^c^After subtracting DPPs due to hypertension treatment in primary prevention.

^d^After subtracting DPPs due to cholesterol lowering treatment in primary prevention.

Abbreviations: DPP, Death prevented or postponed, secq, socio-economic quintile.

The proportion of DPPs explained by the model differed between secq groups. Better treatment contributed to approximately 25% in the most affluent secq groups and less than 20% in the most deprived group (Table 2). Improvement in risk factors could explain the relative highest proportion for the middle SEC group and less for the most deprived and affluent secq groups. The relative contribution from reduction in cholesterol level (minus treatment) was highest in the most deprived quintile compared to the most affluent quintile, whereas for smoking and hypertension there was no consistent difference between the five secq groups (Table 3).

Substituting risk factor data from cohort studies (CCHS 3 and 4) to data from survey studies (Dan MONICA III and Health 2006) lead to different results for some of the risk factors. Prevalence of smoking was higher in the surveys and could explain 17.6% of DPPS (vs 9.9% in the original model) whereas differences in cholesterol levels and especially systolic blood pressure were lower. Reduction in cholesterol in the surveys could explain 16.5% of DPPs (vs 24.1% in the original model). In the cross sectional surveys blood pressure measurements increased during the study period and therefore contributed to an increase in DPPs (-11.6% vs 6.3% in the original model). Other risk factor estimates were similar, giving a total explanation of 30.6% (vs 40.1% of the original model) of the DPPs. No substantial changes were seen regarding the differences between socioeconomic groups when compared to data from the CCHS cohorts. Data are shown in S1 Technical Appendix.

## Discussion

We found a dramatic reduction in CHD deaths in Denmark from 1991 to 2007, age standardized mortality rates fell by nearly 75% in both men and women. In absolute numbers the decline was most pronounced in the most deprived groups but the relative reduction was generally highest among the most affluent. We found gender differences as the age-standardized death rates in 2007 versus 1991 among the most deprived groups became nearly 1/4 and 1/5 for men and women respectively. Furthermore, whereas male/female ratios were nearly similar in 1991 they became 2 to 1 in 2007. This most likely reflects multiple contributors. However, we found no substantial gender differences in change in treatment uptake or risk factor prevalences. The ratio shift might therefore perhaps be partly explained by risk factors not included in the model. Overall, he IMPACTsec model could explain approximately 64% of the DPPs with the biggest contribution attributable to improvements in the prevalence of risk factors.

Approximately two thirds of the explained DPPs resulted from contribution from risk factors and one third from treatment uptake, much as in previous analyses [5,7,8]. Approximately one third of the CHD mortality reduction could not be explained, perhaps reflecting inaccurate data or risk factors not quantified in the current model.

There have been major improvements in the CHD treatments in the last three decades, including statins, as both primary and secondary prevention. Also, pharmacological improvements of CHF treatment and systematically follow- up for CHF patients in ambulatory clinics throughout Denmark has ensured higher treatment uptakes and persistence to treatment for this patient group. Moreover, coronary revascularization (PCI and CABG) has been widely used in Denmark for both acute treatment for myocardial infarction and elective treatment for angina. These improvements are reflected in our results showing that especially improvements in CHF treatment and higher use of statins contributed to the reduction of CHD mortality.

Almost twice as many deaths were prevented or postponed in the most deprived quintiles compared with the most affluent quintile (1901 DPPs vs 1080 DPPs). However, the mortality rate in the base year was much higher in the most deprived group, especially for women and the relative mortality decline was most pronounced in the most affluent group (79.6% vs 71.1%). This may be explained by a bigger contribution from better treatments in the most affluent. In spite of the fact that the health care system in Denmark is financed through taxes and not depended on private health insurances or self-payment, these findings indicate that even in a collectively financed health care system, the most affluent patients may be better at taking advantage of the treatment offered. This is supported by previous studies showing that the initiation of invasive treatments and preventive pharmacological post-AMI treatment was higher for patients with the highest financial income [11,12]. This may be partly because basic medication costs are not fully covered by the public health insurance system and partly because patients from lower socio-economical groups have more difficulties in sticking to a prolonged medical treatment.

The highest contribution to DPPs among risk factors was reduction in hypercholesterolemia and smoking whose benefits were similar across all socioeconomic groups. The contribution of other risk factors did not show any consistent social gradient. In 2004, Denmark promoted legislation against use of industrial produced trans fatty acids in the food industry and this may have had an indirect beneficial effect on the cholesterol level and also helped to explain the big mortality fall in the most deprived group, assuming that before the ban this group could have had the highest intake of trans fatty acids. Thus, previous studies in UK have indicated that a high consumption of (primarily industrial) trans fatty acids was associated with social disadvantage before a voluntary reformulation of use of industrial trans fatty acids. After reformulation there was a significant reduction of trans fatty acids and the association to social disadvantage [20]. A similar effect may explain the reduction of smoking where a ban was enacted in 2007 but may have had some influence on smoking habits in the preceding years.

Overall, the contribution to DPPs was highest among risk factors. Thus, the contribution of smoking reduction was more than three times that of the overall treatment of myocardial infarction (9.5% vs 2.7%, see Tables 2 and 3). Moreover, since the treatment uptakes in Denmark are high the potential for further reduction in coronary mortality seems more likely to come from further improvements in lifestyle and risk factors than in improvement of treatment uptake. This may also be less expensive and as mentioned above this may also benefit all socioeconomic groups.

This study had many strengths. We used an epidemiologic model (IMPACTsec) that has been applied in a number of countries and found to be valid in explaining the change in coronary mortality. We predominantly used registries with very high quality of individualized data on diagnosis, treatments and financial income and it was possible to merge information on an individual level from different registries using a unique personal code. Thus, we could avoid or minimize the risk of overlap between disease- or treatment groups ensuring that persons only were included in one of the nine mutually independent disease-groups.

Some limitations also need to be acknowledged. First of all, we could not explain one third of the DPPs. Previous IMPACT models used in other countries have explained a higher proportion of the DPPs compared to the Danish IMPACTsec model [4–10]. In our model, however, treatment uptake rates and risk factor decrease was comparable to other countries. The main difference in Denmark compared to previous studies was that the decrease in CHD mortality was so pronounced resulting in lower relative numbers; in other words the Danish IMPACTsec model was not able to explain the extra CHD mortality reduction that was seen in Denmark.” This may be because data of e.g. risk factors or in-hospital treatments were not accurate or because unaccounted factors might have played a role, for example changes in trans-fat intake at the population level In Denmark, we do not have any registry data of community diagnosis and therefore we used data from hospital registries on e.g. DM and angina. Also, we calculated the prevalence in the community of congestive heart failure and number of persons eligible for secondary prevention following ACS from the hospital registry. We assume that most persons with these diagnoses would have had a hospital contact during the previous 11 years. We did not have any nationwide data of in-hospital treatment and therefore performed a survey including 500 randomly chosen patients with myocardial infarction and CHF within one (of five) region of Denmark and assumed that this was representative for the general in-hospital treatment for the whole country. With this limited number there were some of the age-gender-SEC groups that were very small but to optimize the data information we calculated gradients (S1 Technical Appendix). For 1991, we did not have a national prescription registry for medical treatment in the community so we used data from county of Funen and used the same methods as mentioned above. There is no registry with national representative information on the prevalence of risk factors but we used data from a large cohort study with repeating measurements for each decade. This means that we compare nearly the same population which could contribute to a false decline in blood pressure due to higher mortality among those with high blood pressure. We did not include information of other risk factors on e.g. diet, alcohol, trans fatty acids but their effect is partially mediated through some of the risk factors included in the model. Some treatments (CPR and cardiac rehabilitation) were also omitted from this IMPACTsec model but we assume that this may be compensated for by including data on pharmacological treatment or that the differences of treatment uptake was limited between base year and final year. Moreover, the data structure did not allow us to examine interaction or effect modification since data originates from many different surveys and registers, for further details please see S1 Technical Appendix.

Using risk factor data from other sources did reduce the numbers of DPPs explained by the model. This was mainly because these data indicated an increase in mean systolic blood pressure during the study period explaining -11.6% DPPs vs +6.3% in the original model. Other risk factor numbers were more similar. As mentioned above, there may be some selection bias using cohort data since participants with hypertension from the base year may not participate in the final year because of morbidity or mortality. On the other hand blood pressure levels in the surrounding areas/countries usually comparable to Denmark was decreasing in the same period and also the surveys did not have any data from the oldest age group.

## Conclusions

The IMPACT_SEC_ model suggests that the largest contribution to the CHD mortality decline in Denmark from 1991 to 2007 was from improvements in risk factors across all socioeconomic groups. However, we found a clear socioeconomic trend for the treatment contribution favouring the most affluent groups.

## Supporting information

S1 Technical Appendix Technical appendix for the Danish IMPACT_SEC_ Model. (DOCX)
