# Supplementary material for: Explaining trends in coronary heart disease mortality in different socioeconomic groups in Denmark 1991-2007 using the IMPACTSEC model
Source: PLoS One. 2018 Apr 19;13(4):e0194793. doi: 10.1371/journal.pone.0194793 (PMC5909604; doi:10.1371/journal.pone.0194793)
Supplement: S1 Technical Appendix — (DOCX) [file pone.0194793.s001.docx]

**TECHNICAL APPENDIX FOR THE DANISH IMPACT_SEC_ MODEL**

**A M. Joensen^1^, T. Joergensen^2,3,4^, S. Lundbye-Christensen^5^,MB Johansen^5^, M Guzman-Castillo^6^, P Bandosz^6,7^, J Hallas^8^ , EIB. Prescott^9^ S. Capewell^6^** **and M. O'Flaherty^6^.**

**^1^Aalborg University Hospital, Department of Cardiology, Aalborg, Denmark**

**^2^Research Center for Prevention and Health, The Capital Region, Glostrup, Denmark**

**^3^Department of Public Health, Faculty of Health and Medical Sciences, University of Copenhagen, Copenhagen Denmark**

**^4^Faculty of Medicine, Aalborg University, Aalborg, Denmark**

**^5^Unit of Clinical Biostatistics, Aalborg University Hospital, Aalborg, Denmark**

**^6^ University of Liverpool, Department of Public Health and Policy, Liverpool, United Kingdom**

**^7^ Department of Preventive Medicine and Education, Medical University of Gdańsk, Gdańsk, Poland^8^ Clinical Pharmacology, Department of Public Health, University of Southern Denmark, Odense, Denmark**

**^9^Bispebjerg University Hospital, Capital Region of Denmark, Copenhagen, Denmark**

List of contents

[List of abbreviations 3](#_Toc481318836)

[Overview. The IMPACTSEC model: Introduction, detailed methodology and examples of deaths prevented or postponed (DPP) calculations 4](#_Toc481318837)

[1. INTRODUCTION 4](#_Toc481318838)

[2. METHOD AND EXAMPLES 6](#_Toc481318839)

[3. CUMULATIVE RISK-REDUCTION: ADJUSTING DEATHS PREVENTED OR POSTPONED (DPPs) TO CALCULATE CUMULATIVE BENEFIT OF MULTIPLE RISK FACTOR CHANGES 11](#_Toc481318840)

[4. OTHER METHODOLOGICAL CONSIDERATIONS 15](#_Toc481318841)

[Table S1. Population and patientgroup definitions and data sources used in the IMPACT_SEC_ model 21](#_Toc481318842)

[Table S2. Data sources for treatment uptake levels 24](#_Toc481318843)

[Table S3. Risk factors – variable definitions and source 26](#_Toc481318844)

[Table S4. CHD mortality rates 1991 and 2007 by sex and socioeconomic quintiles 29](#_Toc481318845)

[Table S5. Clinical efficacy of interventions: relative risk reductions obtained from meta-analyses, and randomised clinical trials 30](#_Toc481318846)

[Table S6. Case fatality rates for each patient group 35](#_Toc481318847)

[Table S7. Treatment uptake in 1991 and 2007^†^ 36](#_Toc481318848)

[Table S8. Beta coefficients for major risk factors 40](#_Toc481318849)

[Table S9. Relative Risks for CHD used in the IMPACT_SEC_ model for Smoking, Diabetes and Physical Inactivity 42](#_Toc481318850)

[Table S10. Risk factor levels in 1991 and 2007 by sex and socioeconomic quintiles 52](#_Toc481318851)

[(Data from Copenhagen City Heart Study 3 and 4)* 52](#_Toc481318852)

[Table S10a. Risk factor levels in 1991 and 2007 by sex and socioeconomic quintiles (Data from MONICA III and Health 2006)* 53](#_Toc481318853)

[Tables S11. Model fit by age, sex and deprivation quintiles 54](#_Toc481318854)

[Table S12. Uncertainty analysis: parameter distributions, functions and sources 58](#_Toc481318855)

[Annex SA. 75](#_Toc481318856)

[Reference List 77](#_Toc481318857)

| List of abbreviations**:** | |
| --- | --- |
| ACE Inhibitors | Angiotensin-converting enzyme inhibitors |
| ACS | Acute coronary syndrome |
| AMI/MI  AR | Acute myocardial infarction  Additive risk-reduction |
| ARB | Angiotensin receptor blocker |
| ATC | Anatomical Therapeutic Chemical Classification System |
| BMI | Body mass index |
| CABG | Coronary artery bypass graft |
| CAD | Coronary artery disease |
| CCHS | Copenhagen City Heart Study |
| CFR | Case fatality rates |
| CHD/CVD | Coronary heart disease/Cardiovascular diesease |
| CPR number  CR | Civil personal registry number (a unique personal 10-digit code)  Combined/cumulative risk-reduction |
| CPS-II | Cancer Society Prevention Study II |
| DECODE | Diabetes Epidemiology: Collaborative Analyses of Diagnostic Criteria in Europe |
| DG | Disease group |
| DM | Diabetes mellitus |
| DNPR | Danish National Patient Registry |
| DPP | Deaths prevented or postponed |
| EU | European Union |
| GBD | Global burden of disease |
| HF | Heart failure |
| ICD-8 (10) | International classification of diseases version 8 (10) |
| NSTEACS | Non-ST elevation acute coronary syndrome |
| NSTEMI | Non-ST elevation myocardial infarction |
| OR | Odds ratio |
| P | Prevalence |
| PARF | Population attributable risk fraction |
| PCI | Percutaneous coronary intervention |
| PG IIB/IIIA | Platelet glycoprotein IIB/IIIA inhibitors |
| RR | Relative risk |
| RRR | Relative risk reduction |
| SBP | Systolic blood pressure |
| SE | Standard error |
| SEC | Socio-economic |
| SECQ | Socio-economic quintile |
| STEMI | ST-elevation myocardial infarction |
| UA  WHO | Unstable angina  World Health Organization |

# Overview. The IMPACTSEC model: Introduction, detailed methodology and examples of deaths prevented or postponed (DPP) calculations

# INTRODUCTION

IMPACT is a deterministic, cell-based policy model. It uses epidemiological information to estimate the contributions of population-level risk factor changes (impacting mainly on incidence) and changes in the uptake of evidence-based treatments (impacting mainly on case fatality) between two points in time (the start-year and the end-year). The primary outcome measure of the model is the deaths prevented or postponed (DPPs).

The starting point for the model is to calculate the ‘target’ number of deaths the model needs to explain. This target number is obtained by using death counts recorded in the official registration system to calculate the difference between the actual observed Coronary Heart Disease (CHD) deaths recorded in the end-year from expected deaths, i.e. simple indirect standardisation: the number that would have occurred in the end-year had the CHD mortality rates remained the same as in the start-year.

The calculation of the modelled estimate of DPPs rests on utilising two well-studied relationships: firstly, that between risk factor change and the relative reduction in CHD mortality; secondly, that between treatment uptake and reductions in case-fatality in patients with a specific form of CHD.

The model applies the relative risk reduction quantified in previous randomised controlled trials and meta-analyses to estimate the mortality reduction attributable to:

a) temporal change in risk factor prevalence (in those without diagnosed CHD) to calculate the DPPs ‘explained’ by specific risk factor trends;

b) net change over the period in the uptake of specific treatments in patients with each specific form of CHD to estimate DPPs ‘explained’ owing to improved 1-year case fatality rates. Great care is taken to avoid double counting the same individuals.

The mortality benefits from the risk factor reduction in the population, and the treatment benefits in patient groups are then summed. Thus summing uses a cumulative approach (rather than an additive approach), in order to avoid double-counting of benefits in the same individual. (This approach is detailed in Section 3.1).

This mortality sum represents the deaths prevented or postponed (DPPs) ‘explained’ by the model.

At the end of the modelling process, the total DPPs ‘explained’ by the model is then compared with the observed fall in deaths (the ‘target’ to be explained).

**Model fit** is therefore calculated as the difference between the observed deaths and model DPPs, and expressed as the percentage explained. This measures the extent to which the model was successful in explaining the observed change in CHD mortality in the population.

A policy model like IMPACT thus stands in contrast to a typical multivariate regression model. A typical multivariate regression model represents a statistical approach to describing a single data-set, for instance generated by a single cohort or RCT. In contrast, a policy model such as IMPACT seeks to integrate and synthesise best estimates from a variety of sources to reliably estimate the extent to which a range of factors, acting in combination, explain or predict an outcome. We did not obtain the parameters for this model by running regressions. Rather, the model incorporates the best coefficients from the largest meta-analysis or randomised controlled trials of the reduction in case fatality attributed to treatment or the independent effect sizes of a unit change in each risk factor on CHD mortality.

Examples of the calculation method used for estimating the DPPs due to treatment uptake (Example 1) and for continuous and binary risk factor change (Examples 2 and 3) are provided below. Earlier versions of the IMPACT mortality model have been previously applied to national data from Europe, United States, Ontario, New Zealand and China.(1-5) The methodology has previously been described in detail online and elsewhere.(1,2,4)

***The IMPACT_SEC_ model***

We have now extended the IMPACT model to accommodate sub-national variation in CHD mortality trends by socioeconomic circumstances (IMPACT_SEC_ model). In the Danish IMPACT_SEC_ model we used information from Statistic Denmark on financial income for the years before the index years (1990 and 2006) for persons alive and living in Denmark 1. of January 1991 and 2007, respectively. Based on this information the Danish population was divided into socio-economic quintiles (secq) (groups 1-5) as a proxy indicator of socioeconomic circumstances. The distribution into quintiles was based on age- and gender-specified data so we had equally sized quintiles for each age and gender group. In Denmark each citizen has a unique 10 digit personal identification number (civil personal registry number (CPR number)) that can be used to merge information from different registries (e.g. diagnosis, treatment and financial income) on an individual level. This model examines the effects of changes in treatment uptake and risk factor trends on changes in mortality from coronary heart disease (CHD) among adults in Denmark aged 25 – 84 years, stratified into equal quintiles by population size. The tables included in this supplementary appendix provide details about the sources and methods that were used.

# METHOD AND EXAMPLES

**2.1 Changes in mortality rates from CHD, Denmark 1991 to 2007**

Data sources used in examining the changes in CHD mortality rates over 1991 to 2007 are shown in Table S1. Mortality rates from CHD were calculated using the underlying cause of death (*1991*: ICD8 410-414 and 429; *2007*: ICD10 I20-I25). The indirect method of age-standardisation was used with the Danish projected 1991 population as the reference population.

**2.2** Expected and observed number of deaths from CHD

Data sources used to estimate the observed and expected number of deaths from CHD for 1991 and 2007 are shown in Table S1. The expected number of CHD deaths in 2007 was calculated by multiplying the age-sex-sec quintile specific mortality rates from CHD in 1991 by the population counts for 2007 in that age-sex-sec quintile stratum. Summing over all strata then yielded the expected number of deaths. The difference between the number of expected and observed deaths from CHD represented the mortality fall, the total number of DPPs from the combined changes in treatment uptake and risk factor levels between two cross-sectional time points.

**2. 3** Treatment component of IMPACT_SEC_ model

The treatment component of the IMPACT_SEC_ model included nine mutually exclusive CHD patient groups:

- DG1: Patients discharged from hospital with the diagnoses acute ST-elevation myocardial infarction (STEMI) within 1 year.
- DG2: Patients discharged from hospital with the diagnoses unstable angina (UA) or non-ST elevation acute coronary syndrome (NSTEMI) within 1 year.
- DG3: Community-dwelling patients who have survived a myocardial infarction for over a year
- DG4: Patients who have undergone a revascularisation procedure the pevious 11 years: Coronary Artery Bypass Grafting (CABG), or a Percutaneous Coronary Intervention (PCI)
- DG5: Community-dwelling patients with discharged with the diagnoses angina pectoris within the previous 11 years
- DG6: Patients discharged from hospital with heart failure (HF) (due to CHD) within 1 year.
- DG7: Community-dwelling patients with heart failure (due to CHD)
- DG8: Hypercholesterolaemic subjects without CHD eligible for cholesterol lowering therapy such as statins
- DG9: Hypertensive individuals without CHD eligible for anti-hypertensive therapy

If a person had more than one cardiac diagnosis they were categorized into only one disease-group representing the group with the highest mortality risk, where hospital admission for CHF (DG6) was considered as the highest risk followed by CHF in the community (DG7), admission for STEACS (DG1), NSTEACS (DG2), previous ACS (DG3), previous CABG or PCI (DG4), angina pectoris (DG5), patients eligible for primary prevention for hypercholesterolemia (DG8) or hypertension (DG9). By using the personal 10 digit code we could exclusively categorize each patient into one disease-group. In contrast to other IMPACTsec models we therefore had no overlaps between disease-groups.

STEMI NSTEMI patients were examined separately as the management and outcomes of these entities differ markedly.(6) The data sources used to estimate the size of each treatment group (stratified by age-sex-secq) are shown in Table S1. For each group, we estimated the number of DPPs that were attributable to various treatments. A list of the treatments considered in the model and the data sources used to estimate the percentage of patients receiving treatments is shown in Table S2.

The general approach to calculate the number of DPPs from an intervention among a particular patient group was first to stratify by age, sex and secq; then to multiply the estimated number of patients in 2007 in turn by the proportion of these patients receiving a particular treatment, by the one-year case fatality rate, and by the relative reduction in the case fatality rate due to the administered treatment. Sources for treatment uptake are shown in Table S2. Sources for estimates of efficacy (relative risk reductions) are shown in Table S5. We obtained the relative risks based on the most recent published systematic reviews and meta-analyses of epidemiological studies. Each treatment relative risk value in the model was based on a meta-analysis comparison with an older therapy, or in some cases with a placebo if relevant. Age-gender specific case fatality rates for each patient group are presented in Table S6.

It was assumed that compliance (concordance), the proportion of treated patients actually taking therapeutically effective levels of medication, was 100% among hospital patients, 70% among symptomatic community patients, and 50% among asymptomatic community patients taking lipid-lowering drugs or anti-hypertensive medication for primary prevention.

**Example 1: Estimation of DPPs from a specific treatment**

*Mortality fall in STEMI patients as a result of taking aspirin in men aged 55-64 in the most deprived quintile*

For example, in 2007, 147 men aged 55-64 in the most deprived quintile were hospitalised with STEMI (ICD10: I21.0-3). Uptake of aspirin was estimated to be 100%. Aspirin use reduces case fatality in patients with ST-segment elevation by approximately 23%. The underlying one-year case fatality rate in these men was approximately 6%. The DPPs for at least a year were therefore calculated as:

***Patient numbers × treatment uptake × relative mortality reduction × one year case fatality***

= 147× 100% × 23% × 6% ≈ 2 DPPs

This calculation was then repeated:

1. For each age-gender-secq group (60 in total).
2. Incorporating a Mant and Hicks adjustment for multiple medications within each patient group (see below).

**2.4** Risk factor component of IMPACT_SEC_ model

The second part of the IMPACT_SEC_ model estimated the number of DPPs related to changes in cardiovascular risk factor levels in the population. The risk factors considered were cigarette smoking, total cholesterol, systolic blood pressure, body mass index, diabetes mellitus (DM), and physical inactivity. Information from cohort studies (Copenhagen City Heart Study (CCHS) 3 and 4)(7) was used to calculate trends in the prevalence (or mean values) of each risk factor (Table S3 and Annex SA). To control for possible selection bias regarding data collection from the cohort studies, for sensitivity analyses we also substituted estimates for risk factors fraction obtained from the CCHS cohort studies with estimates from cross sectional population surveys Dan-MONICA III (1991) and Health 2006 (2006-7)(8). Two approaches to calculate DPPs from changes in risk factors were used: the regression approach and change in the Population Attributable Risk Fraction (PARF). These are illustrated below.

*Estimating DPPs from risk factor change – regression approach for continuous risk factors*

In the regression approach – used for systolic blood pressure (SBP), total cholesterol, and body mass index (BMI)– the number of CHD deaths in 1991 (the start year) after adjusting for population change between 1991 and 2007 were multiplied by the absolute change in risk factor level, and by a regression coefficient (‘beta’) quantifying the estimated change in CHD mortality that would result from a one-unit change in risk factor level (Table S8). Natural logarithms were used, as is conventional, in order to best describe the log-linear relationship between changes in risk factor levels and mortality. For example, using the meta-analysis by Lewington et al(9) a beta coefficient of -0.431 (for women 55-64 years) corresponds to an estimated 35% reduction in CHD mortality rates for a one-unit decrease in the mean cholesterol. Levels of risk factors in 1991 and 2007 by sex and quintile group are shown in Table S10.

**Example 2: Estimation of DPPs from risk factor changes using regression method**

*Mortality fall due to reduction in total cholesterol in women aged 55-64 in the most deprived quintile*

For example, in 1991, there were 124 CHD deaths among 49,789 women aged 55-64 years in the most deprived quintile. The population total had increased to 70,957 in 2007. Applying the CHD death rate from 1991 (248. per 100,000) to the 2007 population gives an (adjusted) total of 176 expected deaths in 2007.

Mean total Cholesterol in this group fell by an estimated 1.28 mMol/l (from 7.04 in 1991 to 5.76 in 2007). The largest meta-analysis reports an estimated age-sex specific reduction in mortality of 35% for every 1 mmol/l reduction in total cholesterol(9), generating a logarithmic coefficient of -0.431 (i.e. natural logarithm of (1 - 0.35)). The subsequent reduction in CHD deaths between 1991 and 2007 was then estimated as the product of three variables:

***DPPs = expected CHD deaths in 2007 (had 1991 mortality rates remained constant) × absolute risk factor reduction between 1991 and 2007 × regression coefficient exponentiated***

DPPs = (1-(exponential (regression coefficient × absolute change))) × expected deaths in 2007

DPPs = (1-(exponential (-0.431 ×1.28 ))) × 176 ≈ 75

This calculation was then repeated for each age-gender-secq group.

Data sources for the number of CHD deaths are shown in Table S1, population surveys CCHS 3 and 4 were used to estimate risk factor trends (Table S3), and sources for the regression (beta) coefficients used in these analyses are listed in Table S5. The regression coefficients were assumed equal across deprivation quintiles.

*Estimating DPPs from risk factor change – PARF approach for binary risk factors*

The PARF approach was used for cigarette smoking, diabetes, and physical inactivity. PARF, which can be interpreted as the proportion by which the mortality rate from CHD would be reduced if the exposure were eliminated(10), was calculated as:

***PARF = [P × (RR - 1)] / [1 + P × (RR - 1)]***

Where P is the prevalence of the risk factor and RR is the relative risk for CHD mortality associated with risk factor presence. A relative risk of 3.3 associated with smoking, for example, expresses the ratio of risk of CHD mortality in smokers to that in non-smokers. DPPs were then estimated as the expected CHD deaths in 2007 (had 1991 mortality rates remained constant) multiplied by the difference in PARF for 1991 and 2007.

**Example 3: Estimation of DPPs from risk factor changes using the PARF method**

*Mortality increase due to increase in diabetes in men aged 65-74 in the most deprived quintile*

For example, the prevalence of diabetes among men in the most deprived quintile aged 65-74 years was 4.3% in 1991 and 8.2% in 2007. Assuming a relative risk of 1.86, the PARF at the national level for men aged 65-74 was 0.036 in 1991 and 0.066 in 2007.

The DPPs attributable to the increase in diabetes prevalence for the most deprived quintile in this male agegroup was therefore:

***DPPs = expected CHD deaths in 2007 (had 1991 mortality rates remained constant) × (PARF_1991_ – PARF_2007_)***

DPPs = expected CHD deaths in 2007 (874) × (0.036 – 0.066) ≈ -26 DPPs

A negative sign for the DPPs denotes deaths increased or brought-forward due to the increase in diabetes prevalence. The calculation was then repeated for each age-gender-secq group.

Relative risks estimated by expert working groups for the World Health Organization’s Global Burden of Disease 2001 Study were used for smoking and physical inactivity.(11) Effect estimates were based on systematic reviews of cohort studies (adjusted for regression dilution bias) and meta-analyses of RCTs. Age-variation in the relative risks for diabetes were taken from the Diabetes Epidemiology: Collaborative Analysis of Diagnostic Criteria in Europe (DECODE) study.(12) These were then applied to the gender-variation in relative risks estimated by Huxley et al.(13) The published relative risk values for smoking, physical inactivity and diabetes are shown in Table S9. These were adjusted in our study to a) match the 10-year age bands and b) employ a dichotomous rather than trichotomous measure of physical activity. Full details on these adjustments are shown below Table S9.

# CUMULATIVE RISK-REDUCTION: ADJUSTING DEATHS PREVENTED OR POSTPONED (DPPs) TO CALCULATE CUMULATIVE BENEFIT OF MULTIPLE RISK FACTOR CHANGES

**3.1** Background

CHD deaths are usually caused by multiple risk factors acting simultaneously. Hence, part of the effect of one risk factor may be mediated through another. For example, physical inactivity may have a direct effect on CHD but may also partly be mediated through its effects on BMI and blood pressure. It is recommended therefore that mortality benefits attributable to risk factors which may be causally related, or which overlap in population groups, should not be combined by simple addition. Ideally, their effects should instead be jointly estimated.(14-18)

We do not currently have sources that allow joint estimation of relative risks for combinations of risk factors in this Danish population. However, several large cohort studies and meta-analyses have published independent risk reduction coefficients for each risk factor included in this study. These are detailed in Tables S8 and S9 for continuous and dichotomous risk factors, respectively. One approach commonly used is to calculate the **cumulative risk-**reduction.(19) This approach accounts for risk factor prevalence overlap but assumes independence of effects.(16,17) The general equation for cumulative risk-reduction is stated as:

Combined (or cumulative) effect (CR) =

1 – ((1-a) × (1-b) × (1-c) ×….× (1-n)) **[1]**

Thus for CHD risk factors, the specific equation is stated as:

**CR** = 1 – ((1-R_SBP_) × (1-R_smoke_) × (1-R_diabetes_) ×….× (1-R_n_))

where R denotes the mortality change attributable to a specific risk factor.

This is in contrast to additive risk-reduction (AR):

**AR** = (R_SBP_) + (R_smoke_) + (R_diabetes_) +…..+ (R_n_) **[2]**

**3.2 Implementation**

For the purposes of this modelling study we first calculated the (additive) DPPs attributed to risk factor change. These were then adjusted down by using the ratio:

Adjustment factor = CR/AR

The adjustment factor would always be expected to be less than 1. In other words, cumulative risk factor reduction would be smaller than the mortality benefits arrived at by a simple summation of the benefits of each risk factor in turn.

The proportional change in the CHD mortality rate between two time points (denoted by R) was calculated using the following formulas(16,17):

Continuous risk factors:

R_continuous_  = 1 – exp(beta × absolute mean risk factor change) **[3]**

Dichotomous risk factors:

R_dichotomous_  = PAF × (ΔP/P) **[4]**

where PAF = [P × (RR - 1)] / [1 + P × (RR - 1)]

and P denotes prevalence at the start-year; RR the relative risk in CHD mortality associated with risk factor presence; and ΔP the change in prevalence between the start and final years.

Formulas [3] and [4] were used to calculate the proportional change in the CHD mortality rate (R) for each risk factor and the steps involved in their estimation are detailed below. However, we made two modifications to the methodology used in previous work(16,17). First, we estimated aggregate change over a sixteen year period (1991-2007) rather than average annual change. Second, additive and cumulative risk-reduction was calculated by using the **absolute** values of R (i.e. disregarding the direction of risk factor change). These are discussed in turn below.

*Calculating aggregate change in risk factors over 1991 and 2007*

Information of risk factors was obtained from population surveys, see above and Table S3. Previous studies(16,17) estimating cumulative risk factor reduction calculated the average annual percentage change in CHD mortality attributable to annual falls in levels of smoking, blood pressure and cholesterol (where annual falls in CHD mortality and risk factor levels were estimated over a specified number of years). Rather than estimate the average annual change over a specific range of years, we were interested in calculating the R values between two fixed points in time (start and end years of the model), sixteen years apart, 1991 and 2007. We therefore adapted formulas [3] and [4], substituting change over the sixteen years study period for the estimation of annual average change.

*Regression models to estimate risk factor change, 1991-2007*

Formulas [3] and [4] require estimates of absolute and relative change in risk factors, respectively. Regression modelling was used to estimate the magnitude of absolute and relative change. See Annex SA.

Estimates of absolute change in the mean levels of risk factors measured on a continuous scale (blood pressure, total cholesterol, and body mass index) were calculated by linear regression. The dependent variable was the risk-factor level for each survey respondent; calendar year (i.e. year of interview) was the explanatory variable entered in the model as a continuous term. Absolute change was measured as the difference between the predicted values for 1991 and 2007, by age and sex.

Estimates of change in prevalence estimates (smoking, diabetes and physical activity) were calculated using a generalised linear model with binomial distribution and a log link function. The outcome variable was binary (1 indicating risk factor presence; 0 absence) with calendar year as the explanatory variable. The absolute difference in predicted values for 2007 and 1991 (ΔP in formula [4]) divided by the 1991 value provided the estimate of relative change.

*Combining risk factors contributing positive and negative benefits to CHD mortality change*

In previous CHD modelling studies(16,17) adjusting for cumulative risk-reduction was straightforward as the trends in smoking, blood pressure and total cholesterol were mostly unidirectional: that is, risk factor levels were declining as were CHD mortality rates. However, in our current modelling study, mean BMI and the prevalence of diabetes increased over 1991-2007 while the four remaining risk factors showed favourable trends. In effect, the impact of risk factor change on CHD mortality was not uniformly beneficial: therefore, the proportional change in CHD mortality attributable to risk factor change was in some cases negative.

In order to avoid positive and negative R values cancelling each other out in the mathematical application of cumulative risk-reduction (formula [2] above), with the perverse effect of the cumulative benefits being apparently greater than the additive in some instances, we first converted all R values into absolute (i.e. sign-free) numbers. We did this on the understanding that the proportional change in CHD mortality associated with risk factor change was independent of the direction of change (e.g. a one unit increase in mean levels of BMI would result in a 3% increase in CHD mortality (for persons<60 years; likewise a one unit fall would result in a 3% fall). This meant that although the R values were not themselves ‘true’ indicators of the total proportional reduction in CHD mortality, they were computed on a like-for-like basis and so the ratio of cumulative to additive (the adjustment factor) risk reduction was an accurate reflection of the degree to which the additive benefits needed to be adjusted down.

Age-gender-sec specific adjustment factors (60 in total) were calculated by taking the ratio of cumulative to additive risk factor reduction. This involved four steps:

1. Regression equations were fitted to individual level survey data to derive the national (Danish) predicted risk factor levels for the start and end years of the model. Regression models were fitted separately by sex and 10 year age bands (from ages 25 to 85). (See section above and Annex SA describing the regression models fitted on the survey data to estimate aggregate risk factor change).
2. R values for continuous risk factors were then calculated using estimates of absolute change over 1991-2007 (formula [3]). R values for dichotomous risk factors were calculated using the estimated PARF in 1991 by the relative change in prevalence (formula [4]). This resulted in 60 R values (for each age-gender-secq group) for each risk factor. All R values were then converted into absolute numbers.
3. The absolute R values were then combined to calculate the additive (AR) and cumulative (CR) risk factor reductions (formulas [1] and [2] respectively). Age-gender-secq specific adjustment factors were then calculated as CR/AR.
4. Multiplying through the age-gender-secq specific DPPs in the model for each risk factor by the corresponding adjustment factor yielded the estimate of the cumulative benefit of risk factor change to CHD mortality decline over the sixteen year period, 1991 and 2007.

# OTHER METHODOLOGICAL CONSIDERATIONS

Other calculations had to be taken into account than change in treatment uptake and risk factors over time, several other adjustments had to be made. These include:

- adjusting the relative reduction in the case fatality rate for persons receiving multiple treatments (poly-pharmacy),
- overlap between pharmacological and non-pharmacological contribution to risk factor change,
- uncertainty analyses,
- measuring net effects,

These are discussed in turn below.

4.1 Accounting for poly-pharmacy

Persons with or at high risk of developing CHD may take a number of different medications. However, data from randomised clinical trials on efficacy of treatment combinations are sparse. Mant and Hicks suggested a method to estimate case fatality reduction by poly-pharmacy.(20) The adjustment is carried out in a step-by-step manner as set out in the example below. First the total effect is calculated using an inappropriate additive model, which is then adjusted using effect size calculation with an appropriate multiplicative model.

**Example 4: Estimation of reduced benefit if patient taking multiple medications (Mant and Hicks approach)**

*Adjustment for polypharmacy in secondary prevention post myocardial infarction in men aged 55-64 in the most deprived quintile*

Taking the example of secondary prevention post myocardial infarction, good evidence (Table S5) suggests that, for each intervention, the relative reduction in case fatality is approximately: aspirin 15%, beta-blockers 23%, angiotensin converting enzyme (ACE) inhibitors 20%, statins 22%, and warfarin 22%. Our best estimates for uptake were respectively 74%, 55%, 44%, 70%, and 1%. .

Assuming a one-year case fatality rate of 0.013 for men aged 55-64 and a total of 2,988 men aged 55-64 residing in the most deprived quintile in 2007 the total DPPs, with no adjustment for poly-pharmacy, would be calculated as shown in the table below:

| **Secondary prevention post MI** | **Numbers** | **Treatment uptake** | **Compliance** | **Relative risk reduction** | **One year case fatality** | **Unadjusted DPPs** |
| --- | --- | --- | --- | --- | --- | --- |
| **Treatment** | **A** | **B** | **C** | **D** | **E** | **(A × B × C × D × E)** |
| Aspirin | 2,988 | 0.74 | 70% | 0.15 | 0.013 | 3.0 |
| Beta blockers | 2,988 | 0.55 | 70% | 0.23 | 0.013 | 3.4 |
| ACE Inhibitors | 2,988 | 0.44 | 70% | 0.20 | 0.013 | 2.4 |
| Statins | 2,988 | 0.70 | 70% | 0.24 | 0.013 | 4.6 |
| Warfarin | 2,988 | 0.01 | 70% | 0.22 | 0.013 | 0.0 |
|  |  |  |  |  |  |  |
| **Total** |  |  |  |  |  | **13.4** |

The Mant and Hicks approach suggests that in individual patients receiving all these interventions, case fatality reduction is very unlikely to be simply additive. Instead, having considered the 15% case fatality reduction achieved by aspirin, the next medication, in this case a beta-blocker, can only reduce the residual case fatality (1-15%). Likewise, the subsequent addition of an ACE inhibitor can then only decrease the remaining case fatality, which will be 1 – [(1-0.15) × (1-0.23)]. The Mant and Hicks approach therefore suggests that a cumulative relative benefit can be estimated as follows:

Cumulative relative benefit = ***1 – [(1 – (uptake of drug A × relative reduction in case fatality rate for drug A)) × (1 – (uptake of drug B × relative reduction in case fatality rate for drug B)) × …. × (1 – (uptake of drug N × relative reduction in case fatality rate for drug N))]***

In considering appropriate treatments for post MI patients, applying relative risk reductions (RRR) for aspirin, beta-blockers, ACE inhibitors, statins, and warfarin then gives the following cumulative relative benefit (using a weighted average of the age-gender-secq specific uptake figures in each quintile):

=1 – [(1 – (aspirin_uptake_ × aspirin_RRR_)) × (1 – (beta blockers_uptake_ × beta blockers_RRR_)) × (1 – (ACE I_uptake_ × ACE inhibitor_RRR_)) × (1 – (statins_uptake_ × statins_RRR_)) × (1 – (warfarin_uptake_ × warfarin_RRR_))

= 1 – [(1 – (0.76 × 0.15)) × (1 – (0.56 × 0.23)) × (1 – (0.45 × 0.20)) × (1 – (0.76 × 0.24)) × (1 – (0.01 × 0.22))]

= 1 – [(0.89) × (0.87) × (0.91) × (0.82) × (1)]

≈ 0.42 (i.e. a 42% lower case fatality)

This represented a 16% relative reduction 1-(0.42/0.50) on the simple additive value of 50%, resulting in 16% fewer DPPs out of an original total of 11.1 DPPs (leaving an adjusted total of 9.1):

***Adjusted DPPs = unadjusted DPPs × (cumulative relative benefit / additive benefit)***

Adjusted DPPs = 13.4 × (0.42/0.51) ≈ 11.3

All treatment DPPs quoted in the results tables refer to the adjusted DPPs.

4.2 Potential overlaps between patient groups: avoiding double counting

There are potential overlaps between CHD patient groups. For example, approximately half the patients having CABG surgery have had a previous myocardial infarction, and approximately 30% of myocardial infarction survivors have or will go on to develop heart failure within 12 months. In the Danish IMPACTsec model, however, we can identify each person in the patient groups by the CPR number and thus avoid including the same person in two different disease groups. We constructed nine non-overlapping patient groups. Following the basic logic of the natural history of coronary disease, patients recorded as having two or more conditions were allocated to the condition that was further along the disease pathway with the highest mortality risk. For instance, if an individual had both chronic angina and heart failure, they were allocated to heart failure.

4.3 Overlap between pharmacological and non-pharmacological contributions to risk factor DPPs

Risk factor improvements, such as lower blood pressure or lower total cholesterol, may be achieved through medications, lifestyle changes, or a combination. In order to separate the DPPs from pharmacological versus non-pharmacological contributions to CHD mortality, we subtracted the DPPs calculated in the treatment (primary prevention) component of the model from the DPPs calculated in the risk factor component. That is, to estimate the impact of population-wide reduction in total cholesterol due to non-pharmacological change, we subtracted the estimated effect of statins for the primary prevention of CHD from the overall number of DPPs due to change in mean total cholesterol. Similarly, to estimate the impact of the population-wide reduction in SBP we subtracted the estimated effect of anti-hypertensive medication for primary prevention from the overall number of DPPs due to change in mean SBP levels.

4.4 Net effects

As some treatments were in use in 1991, the net benefit of an intervention in 2007 was calculated by subtracting the expected number of deaths prevented if the uptake rates in 1991 remained constant from the estimated number of deaths prevented calculated using the 2007 uptake rates

**Example 5: Net effects for treatments**

*Calculating net effects for aspirin use in STEMI cases in men aged 55-64 in the most deprived quintile*

With an estimated total of 147 men aged 55-64 with STEMI in the most deprived quintile, 100% uptake, a relative risk reduction of 23%, a one-year case fatality rate of 34%, and 100% compliance, the total number of DPPs in 2007 was calculated as:

***Patient numbers × treatment uptake_2007_ × compliance × relative mortality reduction × one year case fatality***

= 147 x 100% x 1 x 23% x 34%≈ 11.4 DPPs

Applying the uptake rate in 1991 (14%) gave a total of 1.7DPPs:

***Patient numbers × treatment uptake_1991_ × compliance × relative mortality reduction × one year case fatality***

=147 x 14% x 1 x 23% x 34%≈ 1.7 DPPs

The net DPPs were therefore:

***Net DPPs = DPPs using uptake_2007_ – DPPs using uptake_1991_***

**=11.4 – 1.7=9.7 DPPs**

The estimated changes in treatment uptake between 1991 and 2007 by deprivation quintile are shown in Table S7.

4.5 Uncertainty analyses

We implemented uncertainty analysis in Excel using Ersatz (version 1.0 available at <http://www.epigear.com>). This is an add-on which allows probabilistic bootstrapping in Excel. Ersatz allows repeated random draws from specified distributions for input variables and then calculates the 95% uncertainty intervals from the realised values of the output variable (DPPs). For the Danish IMPACT_SEC_ model, we calculated the uncertainty intervals based on 1000 draws – taking the 95% uncertainty intervals from the 2.5^th^ and 97.5^th^ percentiles. The parameter distributions used for the input variables are shown in Table S12. Worked examples using Ersatz are shown below Table S12.

4.6 Model fit

Overall, the model could not explain 36.0% of the total deaths prevented (i.e. a shortfall of about 4,000 fewer CHD deaths unexplained by the model). However, the percentage unexplained varied by age, gender, and socio-economic circumstances. These are shown in Tables S12.1- S12.4.

4.7 Classification of socio-economic quintiles

Statistic Denmark has registered data on financial income for each citizen in Denmark. We used “epuivalent family disposable income” as a proxy for socio-economic status based on information of “disposable income” (that is financial income registered by the Tax authorities subtracted payment of taxes and interests on loans) and number of persons in the housholds. To compare the disposable income between persons living in households of different sizes the financial income has been adjusted to the size of the household (equivalent family disposable income). If a person lives alone the equivalent family disposable income is the same as the person’s equivalent disposable income.

(For persons living in households > 1 person the adjustment is calculated as follow:

First all disposable financial income for all persons in the household are added together and then this amount is divided by a weighted number of household member: the first adult person (>14 years) counts as one, the following adult persons count 0.5 each and children (<15 years) count 0.3 each.

Example

In a family with two adults and 2 children <15 years with a disposable income of 420,000 Danish Kroner the equivalent family disposable income is 420,000/2.1= 200,000 Danish Kroner. That means that all members of the household will be registered as having an equivalent family disposable income of 200,000 Danish Kroner.

Because information of financial income for persons who died in the index years were not representative for the annual income (because persons did not have any income for the rest of the year after they died) we based our classification of secq-groups on data of financial income for the years previous of the index years, that is 1990 and 2006.

The distribution into quintiles was based on age- and gender-specified data so we had equally sized quintiles for each age and gender group.

# Table S1. Population and patientgroup definitions and data sources used in the IMPACT_SEC_ model

|  |  |
| --- | --- |
| **Information** | **Source** |
|  |  |
|  |  |
| **Population data** | **Data from the different sources were linked on an individual level by using the unique personal 10-digit code (CPR number)** |
| Population counts and number of deaths by age and sex | The Central Office of Civil Registration (the CPR-Office)(21) |
| CHD deaths | Cause of Death Registry  (1991: ICD8 410-414,427)  (2007: ICD10 I20-I25, and I11.0, I13.0, I13.2, I42.0, I42.6, I42.7, I42.9, I50)  It is estimated that approximately half of the HF diagnoses can be ascribed to CHD so we included 50% of the patients with HF death diagnosis (22) |
| **Financial income** | **Statistic Denmark**  For details, see text 4.7 |
| **Diesease groups*** | **The Danish National Patient Registry** (DNPR) |
| **DG6**: Heart failure (HF) admitted to a hospital ward. | *1991*:  ICD8: 427  *2007*:  ICD10: I11.0, I13.0, I13.2, I42.0, I42.6, I42.7, I42.9, I50.0, I50.1 and I50.9. |
| **DG7**: Heart failure (HF) in the community. | *1991*:  ICD8: 427  Diagnosis registered in DNPR in the period 1980-1990 and no hospital admission with a diagnosis of HF (DG6) in 1991.  *2007*:  ICD10: I11.0, I13.0, I13.2, I42.0, I42.6, I42.7, I42.9, I50.0, I50.1 and I50.9.  Diagnosis registered in DNPR in the period 1996-2006 and no hospital admission with a diagnosis of HF (DG6) in 2007 |
| **DG1**: ST elevation Myocardial infarction (STEMI) | *1991*:  MI: ICD8: 410  (The ratio of MI admissions to STEMI and nSTEMI cases taken as 40/60.^13^)  2007:  ICD10: I21.0-3 |
| **DG2**: non STEMI and unstable angina pectoris (UA) | *1991*:  MI: ICD8: 410  (The ratio of MI admissions to STEMI and nSTEMI cases taken as 40/60.^13^)  UAP: ICD8: 411  2007:  NSTEMI: ICD10 I21.4-9  UAP: ICD10: I20.0 |
| **DG3**: Post MI | 1991: ICD8: 410, 411  Diagnosis registered in DNPR in the period 1980-1990.  2007: ICD10: I20.0, I21  Diagnosis registered in DNPR in the period 1996-2006. |
| **DG4**: CABG and PCI | 1991: Procedure code KFN A-F (CABG) or KFNG (PCI) in DNPR in the period 1980 – 1991  2007: Procedure code KFN A-F (CABG) or KFNG (PCI) in DNPR in the period 1996 – 2007 |
| **DG5**: Angina pectoris | 1991: ICD8: 414  Diagnosis registered in DNPR in the period 1980-1991.  2007: ICD10: I20.9 and I25.1.  Diagnosis registered in DNPR in the period 1996-2007. |
| **DG8**: Hypercholesterolemia | **CCHS 3 and 4**(7)**:**  On the basis of Total cholesterol blood tests in population studies (CCHS 3 and 4) we calculated the prevalence of persons with hypercholesterolemia (Total cholesterol>5 mmol/L). The calculations of gradients for gender, age and secq groups are described in detail in Annex SA. We assumed that the prevalence in the population study was representative for the whole nation. |
| **DG9**: Hypertension | **CCHS 3 and 4**(7)**:**  On the basis of blood pressure measurements and questionnaires in population studies (CCHS 3 and 4) we calculated the prevalence of persons with hypertension (systolic blood pressure >140 mmHg or self-reported hypertension). The calculations of gradients for gender, age and secq groups are described in detail in Annex SB. We assumed that the prevalence in the population study was representative for the whole nation. |

* If a person had more than one cardiac diagnosis they were categorized into only one disease-group representing the group with the highest mortality risk, where hospital admission for CHF (DG6) was considered as the highest risk followed by CHF in the community (DG7), admission for STEACS (DG1), NSTEACS (DG2), previous ACS (DG3), previous CABG or PCI (DG4), angina pectoris (DG5), patients eligible for primary prevention for hypercholesterolemia (DG8) or hypertension (DG9). By using the personal 10 digit code we could exclusively categorize each patient into one disease-group. In contrast to other IMPACTsec models we therefore had no overlaps between disease-groups.

Table S2. Data sources for treatment uptake levels

Medical and surgical treatments included in the model

| **Information** | **Source** |
| --- | --- |
| **Medication use in hospital 2007**: | **Survey from region of Northern Jutland** |
| **(DG1 ST-segment elevation myocardial infarction)**:   - Aspirin - Beta Blockers - ACE I or Angiotensin-II receptor antagonists (ARB) - Thrombolysis - Clopidogrel | Survey of medical journals from a random selection of patients (n=500) with STEMI (ICD 10 I21 and verified as having STEMI), admitted to a hospital in the region of Northern Jutland (≈10% of the Danish population) in 2007. |
| **DG2 Non-ST-segment elevation acute coronary syndrome (NSTEACS):**   - Aspirin without heparin - Aspirin & heparin - Platelet glycoprotein IIB/IIIA inhibitors - Beta Blockers - ACE I/ARB - Clopidogrel | Survey of medical journals from a random selection of patients (n=500) with nSTEMI (ICD 10 I21 and verified as having nSTEMI) or unstable angina pectoris (I200), admitted to a hospital in the region of Northern Jutland (≈10% of the Danish population) in 2007. |
| **DG6 Heart failure due to CHD:**   - Aspirin - Beta blockers - ACE I/ARB - Spironolactone | **Danish Medical Agency**  Number of patients who collected prescribed medicine at the pharmacy as registered at the Danish Medical Agency. |
|  |  |
|  |  |
| **Cardiac rehabilitation for MI and revascularisation survivors** | Set to zero, as mentioned in Table S1 |
| **Medication use in the community:**  **Post MI and revascularisation survivors, chronic stable coronary artery disease (CAD), heart failure**   - Aspirin - Beta blockers - ACE I/ARB - Statins - Warfarin - Spironolactone | **Danish Medical Agency**  Number of patients who collected prescribed medicine at the pharmacy as registered at the Danish Medical Agency. |
| **In-hospital and community medication 1991**: | **Prescription registry, Fuen County** |
|  | Clopidogrel, ACE I/ATII, Heparin, Platelet glycoprotein IIB/IIIA inhibitors and Spironalacton were not in use in Denmark in 1991 and treatment uptake was thus set to 0.  We assume that treatment uptakes of beta blockers, Aspirin, antihypertensive and lipid-lowering medication were the same as treatment uptake in the community based on numbers from prescription registry from county of Fuen. Since there were only very sparse data on patients with unstable angina pectoris and heart failure patients in the community we assumed similar treatment uptake as for myocardial infarction and in-hospital treatment for heart failure, respectively.  Thrombolysis uptake was assumed to be 32% based on historical data from 1992(23) |
|  |  |

| **Primary prevention therapies:** | **Danish Medical Agency (2007) and Prescription registry, Fuen County (1991)** |
| --- | --- |
| **Lipid-lowering drugs** | Number of patients who were taking lipid lowering drugs (ATC C10) registered at the Danish Medical Agency (2007) as having collected prescribed medicine at a pharmacy and who never had a hospital admission with a CHD diagnosis (DG1-7) according to DNPR.  In 1991 we only had information of prescription medicine in the county of Fuen and assumed that the uptake of lipidlowering drugs in this population was represantative for the whole nation. |
| **Anti-hypertensive medication** | Number of patients who were taking antihypertensive (at least two of the following: ATC: C02A C02B C02C C02DA C02DB C02DD C02DG C02L C03A C03B C03D C03E C03X C07 C08 C09) registered at the Danish Medical Agency (2007) as having collected prescribed medicine at a pharmacy and who never had a hospital admission with a CHD diagnosis (DG1-7) according to DNPR.  In 1991 we only had information of prescription medicine in the county of Fuen and assumed that the uptake of antihypertensiva in this population was represantative for the whole nation. |
|  |  |
|  |  |
| **Number of patients undergoing revascularisation:** |  |
| **DG1-2 and DG5**: CABG and PCI | **DNPR and Danish Heart Registry**  1991: Procedure code KFN A-F (CABG) or KFN G (PCI) in DNPR  2007: Procedure code KFN A-F (CABG) or KFN G (PCI) in DNPR or registered with either CABG or PCI procedures in the Danish Heart Register |

# Table S3. Risk factors – variable definitions and source

Information on risk factors have been achieved through population surveys: Copenhagen City Heart Study 3 and 4 (CCHS)

CCHS has previously been described in details.(7) Briefly, it is a cohort study including over 20.000 men and women randomly drawn from Copenhagen Population Register and invited to participate with the purpose to describe the distribution of cardiovascular risk factors in the population and to examine these risk factors relation to morbidity and mortality. The first sample was drawn in 1976 and was age-stratified within 5-year age groups for persons over 20 years with main emphasis on the age groups from 35 to 70 years. At the following examination in 1981-83, 1991-94 and 2002-3 the study population was supplemented with the youngest agegroups while all previous participants alive and living in Denmark were invited. All subjects filled in a self administered questionnaire (concerning e.g. symptoms and diseases (DM), education and socio-economic status, smoking habits and physical inactivity), had a physical examination (BMI, blood pressure), and had a non-fasting venous blood sample (lipids).

When we only had risk factors measurements for 1993 and 2003 we calculated gradients to estimate values for 1991 and 2007, as described in Annex SA.

| **Risk factor** | **Survey years** | **Description** |
| --- | --- | --- |
| **Current cigarette smoking** | 1993 and 2003 | Self-reported status |
| **SBP (mmHg)** |  | Calculated as the mean of the 2^nd^ and 3^rd^ readings for those who had not eaten, consumed alcohol or smoked in the 30 minutes prior to measurement. Those reporting taking blood pressure lowering drugs were included |
| **Body Mass Index** |  | Weight (kg) divided by height squared (m^2^) for all respondents with valid height and weight measurements. |
| **Total cholesterol (mmol/l)** |  | P-Cholesterol>5 mMol/L. Those reporting taking lipid lowering drugs were included |
| **Diabetes mellitus** | 1980-1991 and 1996-2007 | Patients registered in DNPR with a diagnosis of DM (ICD8 250) from 1980-1991 and 1996-2007 (ICD10 E10.x or E11.x) |
| **Physical inactivity** | 1993 and 2003 | Persons who did not spend 30 minutes or more of moderate or vigorous activity on at least five days per week. Occupational activity was excluded. |
|  |  |  |

# Table S4. CHD mortality rates 1991 and 2007 by sex and socioeconomic quintiles

|  | **Year** | **Denmark** | **SECQ1** | **SECQ2** | **SECQ3** | **SECQ4** | **SECQ5** |
| --- | --- | --- | --- | --- | --- | --- | --- |
| **Male** |  |  |  |  |  |  |  |
| **Population (000s)** | **1991** | 1555 | 311 | 311 | 311 | 311 | 311 |
|  | **2007** | 1700 | 340 | 340 | 340 | 340 | 340 |
| **Observed CHD deaths** | **1991** | 8272 | 1098 | 1425 | 1587 | 1890 | 2273 |
|  | **2007** | 2421 | 253 | 392 | 434 | 598 | 745 |
| **Age-standardised rate (100,000)** | **1991** | 532 | 353 | 458 | 510 | 608 | 730 |
|  | **2007** | 138 | 73 | 114 | 126 | 170 | 208 |
| **Expected deaths^††^** | **2007** | 9396 | 1239 | 1610 | 1801 | 2166 | 2580 |
| **DPPs^‡^** | **2007** | 6975 | 986 | 1219 | 1367 | 1568 | 1836 |
| **% of expected deaths prevented** | **2007** | 74.2 | 79.6 | 75.7 | 75.9 | 72.4 | 71.1 |
| **Female** |  |  |  |  |  |  |  |
| **Population (000s)** | **1991** | 1647 | 329 | 329 | 329 | 329 | 329 |
|  | **2007** | 1760 | 352 | 352 | 352 | 352 | 352 |
| **Observed CHD deaths** | **1991** | 5597 | 741 | 889 | 983 | 1072 | 1913 |
|  | **2007** | 1462 | 158 | 253 | 330 | 340 | 381 |
| **Age-standardised rate (100,000)** | **1991** | 340 | 225 | 270 | 298 | 325 | 581 |
|  | **2007** | 89 | 48 | 77 | 100 | 103 | 116 |
| **Expected deaths^†^** | **2007** | 5575 | 728 | 882 | 992 | 1083 | 1890 |
| **DPPs^‡^** | **2007** | 4135 | 575 | 638 | 669 | 744 | 1509 |
| **% of expected deaths prevented** | **2007** | 74.1 | 79.0 | 72.3 | 67.5 | 68.7 | 79.8 |
| **Total** |  |  |  |  |  |  |  |
| **Population (000s)** | **1991** | 3202 | 641 | 641 | 641 | 641 | 641 |
|  | **2007** | 3461 | 692 | 692 | 692 | 692 | 692 |
| **Observed CHD deaths** | **1991** | 13879 | 1840 | 2316 | 2572 | 2964 | 4187 |
|  | **2007** | 3870 | 408 | 638 | 759 | 939 | 1128 |
| **Age-standardised rate (100,000)** | **1991** | 433 | 287 | 361 | 402 | 463 | 654 |
|  | **2007** | 112 | 60 | 95 | 112 | 136 | 161 |
| **Expected deaths^†^** | **2007** | 14981 | 1969 | 2494 | 2794 | 3251 | 4473 |
| **Total DPPs^‡^** | **2007** | 11111 | 1561 | 1857 | 2036 | 2313 | 3345 |
| **% of expected deaths prevented** | **2007** | 74.2 | 79.3 | 74.4 | 72.9 | 71.1 | 74.8 |

^†^ Expected deaths = CHD deaths expected in 2007 had 1991 CHD rates remained.

^‡^ DPPs, deaths prevented or postponed. DPPs = expected – observed deaths in 2007

# Table S5. Clinical efficacy of interventions: relative risk reductions obtained from meta-analyses, and randomised clinical trials

| **Treatments** | **Relative risk reduction^†^** | **Comments** | **Source paper: First author (year), notes** |
| --- | --- | --- | --- |
| ***ST elevation myocardial infarction (STEMI)*** | | | |
|  |  |  |  |
| **Thrombolysis** | 31% (95% CI: 14,45) | <55 years: Odds Ratio (OR)=0.692; Relative Risk Reduction (RRR)=30.8% (95% CI: 14,45)  55-64 years: OR=0.736; RRR=26.4% (95% CI: 17,40)  65-74 years: OR=0.752; RRR=24.8% (95% CI: 15,37)  > 75 years: OR=0.844; RRR=15.6% (95% CI: 4,30) | Estess (2002)(24) |
| **Aspirin** | 23% (95% CI: 15,30) | RRR=23% (95% CI: 15,30): outcome is vascular deaths | ISIS-2 (1988)(25) |
| **Primary CABG surgery** | 39% (95% CI: 23,52) | OR=0.61 (95% CI: 0.48,0.77); RRR=39% (95% CI: 23,52) on page 565, 0-5 year mortality | Yusuf (1994)(26) |
| **Primary PCI** | 30% (95% CI: 15,42) | OR=0.70 (95% CI: 0.58,0.85); RRR=30% (95% CI: 15,42) outcome compares primary angioplasty to thrombolytics. | Keeley (2003)(27) |
| **Beta blockers** | 4% (95% CI: -8,15) | OR=0.96 (95% CI: 0.85,1.08); RRR=4% (95% CI: -8,15) on page 1732 | Freemantle (1999)(28) |
| **ACE inhibitors** | 7% (95% CI: 2,11) | OR=0.93 (95% CI: 0.89,0.98); RRR=7% (95% CI: 2,11) for 30 day mortality in myocardial infarction | ACE Inhibitor Myocardial Infarction Collaborative Group (1998)(29) |
| **Clopidogrel** | 3% (95% CI: 1,6) | RRR=3% (95% CI: 1,6) for 30 day mortality in myocardial infarction | Chen (2005) Sabatine 2005 (30,31) |
|  |  |  |  |
| ***Non-ST-segment elevation acute coronary syndrome (NSTEACS):*** | | | |
|  |  |  |  |
| **Aspirin alone** | 15% (95% CI: 11,19) | OR=0.85 (95% CI: 0.49,0.95); RRR=15% (95% CI: 11,19). Outcome is vascular and nonvascular deaths on page 75. Assume appropriate for patients with NSTE-ACS. | Antithrombotic Trialists’ Collaboration (2002)(32) |
| **Aspirin & heparin** | 33% (95% CI: -2,56) | OR=0.67 (95% CI: 0.48,1.02); RRR=33% (95% CI: -2,56%) in Table 2. The study outcome is composite MI death and non-fatal MI; compares those on aspirin & heparin to aspirin only. | Oler (1996)(33) |
| **Platelet glycoprotein IIB/IIIA inhibitors** | 9% (95% CI: 2,16) | OR=0.91 (95% CI: 0.84,0.98); RRR=9% (95% CI: 2,16). Study looked at acute coronary syndrome without persistent ST elevation. | Boersma (2002)(34) |
| **Early PCI** | 32% (95% CI: 5,51) | OR=0.68 (95% CI: 0.49,0.95); RRR=32% (95% CI: 5,51) | RITA 3 (Fox 2005)(35) |
| **Primary CABG surgery** | 39% (95% CI: 23,52) | OR=0.61 (95% CI: 0.48,0.77); RRR=39% (95% CI: 23,52) on page 565, 0-5 year mortality | Yusuf (1994)(26)  Assumed similar as STEMI. |
| **Clopidogrel** | 7% (95% CI: 2,11) | RRR=7% (95% CI: 2,11) | Yusuf (2001)(36) |
| **Beta blockers** | 4% (95% CI: -8,15) | OR=0.96 (95% CI: 0.85,1.08); RRR=4% (95% CI: -8,15) on page 1732 | Freemantle (1999)(28)  Assumed similar as STEMI. |
| **ACE inhibitors** | 7% (95% CI: 2,11) | OR=0.93 (95% CI: 0.89,0.98); RRR=7% (95% CI: 2,11) for 30 day mortality in myocardial infarction | ACE Inhibitor Myocardial Infarction Collaborative Group (1998)(29) |
| ***Secondary prevention post myocardial infarction/revascularisation:*** | | | |
|  |  |  |  |
| **Aspirin** | 15% (95% CI: 11,19) | OR=0.85 (95% CI: 0.49,0.95); RRR=15% (95% CI: 11,19). Outcome is vascular and nonvascular deaths on page 75. This data seems to be appropriate to this outcome in CHD patients. | Antithrombotic Trialists’ Collaboration (2002)(32) |
| **Beta blockers** | 23% (95% CI: 15,31) | OR=0.77 (95% CI: 0.69,0.85); RRR=23% (95% CI: 15,31) on page 1734. Odds of death in long term trials. | Freemantle (1999)(28) |
| **ACE inhibitors or Angiotensin-II receptor antagonists** | 20% (95% CI: 13,26) | OR=0.80 (95% CI: 0.74,0.87); RRR=20% (95% CI: 13,26) on page 1577, death up to four years [endpoint of study looking at those with heart failure or LV dysfunction]. | Flather (2000)(37) |
| **Statins** | 24% (95% CI: 10,26) | RRR=24% (95% CI: 10,26)  Intensive statin therapy in acute coronary syndromes. | Hulten (2006)(38) |
| **Warfarin** | 22% (95% CI: 13,31) | OR=0.78 (95% CI: 0.67,0.90); RRR=22% (95% CI: 10,33) | Anand and Yusuf (1999)(39) |
|  |  |  |  |
| ***Chronic stable coronary artery disease:*** | | | |
|  |  |  |  |
| **CABG surgery** | 39% (95% CI:23,52) | OR = 0.61 (95% CI: 0.48-0.77), RRR 39% (95% CI: 23,52) on page 565, 5 year mortality | Yusuf (1994)(26) |
|  |  |  |  |
| **Angioplasty** | No effect |  | Boden (2007)(40) |
| **Aspirin** | 15% (95% CI: 11,19) | OR=0.85 (95% CI: 0.49-0.95); RRR=15% (95% CI: 11,19). Outcome is vascular and nonvascular deaths on page 75. | Antithrombotic Trialists’ Collaboration (2002)(32) |
| **Statins** | 23% (95% CI: 10,26) | RRR=23% (95% CI 10,26)  Standard dose statin therapy in coronary artery disease. | Wilt (2004)(41) |
| **ACE inhibitors/ARB** | 17% (95% CI: 6,28) | RRR=17% (95% CI 6,28) | Al-Mallah (2006)(42) |
| ***Heart failure in patients requiring hospitalisation or in the community:*** | | | |
|  |  |  |  |
| **ACE inhibitors** | 20% (95% CI: 13,26) | OR=0.80 (95% CI: 0.74,0.87); RRR=20% (95% CI: 13,26) on page 1577 [death up to four years was study endpoint for those with heart failure or LV dysfunction] | Flather (2000)(37) |
| **Beta blockers** | 35% (95% CI: 26,43) | OR=0.65 (95% CI: 0.57,0.74); RRR=35% (95% CI: 26,43): all cause mortality | Shibata (2001)(43) |
| **Spironolactone** | 30% (95% CI: 18,41)  31% (95% CI: 18,42) | OR=0.70 (95% CI: 0.59,0.82); RRR=30% (95% CI: 18,41) in those that had at least one cardiac related hospitalisation.  OR=0.69 (95% CI: 0.58,0.82); RRR=31% (95% CI: 18,42) in entire study population consisting of those with community heart failure, page 711. | Pitt (1999)(44) |
| **Aspirin** | 15% (95% CI: 11,19) | OR=0.85 (95% CI: 0.49,0.95); RRR=15% (95% CI: 11,19). Outcome is vascular and nonvascular deaths on page 75. | Antithrombotic Trialists’ Collaboration (2002)(32) |
| **Statins** | No effect |  | Kjekshus (2007) and Tavazzi (2008) (45,46) |
| ***Primary prevention therapies:*** | | | |
|  |  |  |  |
| **Treatments for high blood pressure** | 13% (95% CI: 6,19) | OR=0.87 (95% CI: 0.81,0.94); RRR=13% (95% CI: 6,19) in those with high blood pressure without disease at entry. [RRR=29% (95% CI: 17,37) those with average blood pressure and CHD, treated with ACE inhibitors] | Law (2003)(47) |
| **Statins** | 35% (95% CI: 11,52) | OR=0.65 (95% CI: 0.48,0.89); RRR=35% (95% CI: 11,52) for CHD mortality (only trials using statins), Figure 3 on page 4 | Pignone (2000)(48) |

^†^Relative risk reduction (RRR) calculated as 1 – odds ratio

# Table S6. Case fatality rates for each patient group

| **Patient group** | **AMI** | **POST AMI** | **Acute coronary syndrome (ACS)** | **Post revascularisation** | **Chronic stable coronary artery disease** | **Heart failure in hospital** | **Heart failure in community** | **Hypertension** | **Hyper-cholesteraemia** |
| --- | --- | --- | --- | --- | --- | --- | --- | --- | --- |
| **Interval** | **30 day** | **1 year** | **1 year** | **1 year** | **1 year** | **1 year** | **1 year** | **1 year** | **1 year** |
| **Men:** |  |  |  |  |  |  |  |  |  |
| **25-34** | 0.03 | 0.009 | 0.01 | 0.250 | 0.006 | 0.14 | 0.04 | 0.000 | 0.000 |
| **35-44** | 0.02 | 0.006 | 0.01 | 0.050 | 0.009 | 0.14 | 0.04 | 0.001 | 0.001 |
| **45-54** | 0.03 | 0.006 | 0.02 | 0.020 | 0.012 | 0.13 | 0.06 | 0.002 | 0.002 |
| **55-64** | 0.06 | 0.013 | 0.03 | 0.030 | 0.016 | 0.22 | 0.08 | 0.006 | 0.006 |
| **65-74** | 0.16 | 0.027 | 0.05 | 0.045 | 0.029 | 0.34 | 0.13 | 0.014 | 0.014 |
| **75-84** | 0.34 | 0.067 | 0.11 | 0.078 | 0.065 | 0.44 | 0.20 | 0.035 | 0.035 |
| **85+** | 0.51 | 0.189 | 0.26 | 0.194 | 0.163 | 0.61 | 0.32 | 0.094 | 0.094 |
|  |  |  |  |  |  |  |  |  |  |
| **Women:** |  |  |  |  |  |  |  |  |  |
| **25-34** | 0.03 | 0.008 | 0.01 | 0.000 | 0.007 | 0.50 | 0.05 | 0.000 | 0.000 |
| **35-44** | 0.05 | 0.008 | 0.01 | 0.000 | 0.007 | 0.17 | 0.05 | 0.001 | 0.001 |
| **45-54** | 0.06 | 0.011 | 0.02 | 0.033 | 0.010 | 0.06 | 0.05 | 0.002 | 0.002 |
| **55-64** | 0.11 | 0.014 | 0.02 | 0.044 | 0.014 | 0.24 | 0.08 | 0.004 | 0.004 |
| **65-74** | 0.18 | 0.028 | 0.05 | 0.064 | 0.025 | 0.31 | 0.12 | 0.014 | 0.014 |
| **75-84** | 0.30 | 0.052 | 0.10 | 0.084 | 0.054 | 0.39 | 0.17 | 0.035 | 0.035 |
| **85+** | 0.49 | 0.177 | 0.19 | 0.083 | 0.155 | 0.37 | 0.30 | 0.094 | 0.094 |
|  |  |  |  |  |  |  |  |  |  |

**Source:** Wijeysundera et.al (2010)^5^

# Table S7. Treatment uptake in 1991 and 2007^†^

|  | **Denmark** |  | | | **SECQ1** | | | | | | **SECQ2** | | | | **SECQ3** | | | | | **SECQ4** | | | | | | **SECQ5** | | |  |  |
| --- | --- | --- | --- | --- | --- | --- | --- | --- | --- | --- | --- | --- | --- | --- | --- | --- | --- | --- | --- | --- | --- | --- | --- | --- | --- | --- | --- | --- | --- | --- |
|  | **N** | **Uptake (%)** | | | **N** | | **Uptake (%)** | **N** | | **Uptake (%)** | | | | **N** | | **Uptake (%)** | | | **N** | | **Uptake (%)** | | | **N** | **Uptake (%)** | | | | | |
|  |  | **1991** | **2007** |  | | **1991** | **2007** | |  | | | **1991** | **2007** | |  | | **1991** | **2007** | |  | | **1991** | **2007** |  | | | **1991** | **2007** | |  |
| ***ST elevation myocardial infarction (STEMI):*** | | | | | | | | | | | | | | | | | | | | | | | | | | | | |  |  |
| Thrombolysis | 2,833 | *24.1* | 0 | 435 | | 22.8 | 0 | | 533 | | | 23.7 | 0 | | 562 | | 23.9 | 0 | | 653 | | 24.6 | 0 | 651 | | | 24.9 | 0 | |  |
| Aspirin | 2,833 | *12,4* | 91.1 | 435 | | 12.4 | 91.1 | | 533 | | | 12.5 | 91.3 | | 562 | | 12.4 | 90.4 | | 653 | | 12.5 | 91.1 | 651 | | | 12.4 | 91.1 | |  |
| B-Blocker | 2,833 | *14.9* | 87.7 | 435 | | 14.3 | 84.9 | | 533 | | | 14.9 | 83.3 | | 562 | | 14.6 | 85.2 | | 653 | | 15.2 | 88.7 | 651 | | | 15.2 | 94.0 | |  |
| ACE I/ARB | 2,833 | *0* | 51.6 | 435 | | 0 | 57.6 | | 533 | | | 0 | 58.5 | | 562 | | 0 | 56.1 | | 653 | | 0 | 49.6 | 651 | | | 0 | 40.1 | |  |
| Clopidogrel | 2,833 | *0* | 91.7 | 435 | | 0 | 88.3 | | 533 | | | 0 | 94.0 | | 562 | | 0 | 96.0 | | 653 | | 0 | 93.3 | 651 | | | 0 | 86.3 | |  |
| Primary PCI | 2,833 | *0* | 63.7 | 435 | | 0 | 62.1 | | 533 | | | 0 | 61.3 | | 562 | | 0 | 62.8 | | 653 | | 0 | 66.6 | 651 | | | 0 | 64.4 | |  |
| Primary CABG | 2,833 | *0* | 4.1 | 435 | | 0 | 3.7 | | 533 | | | 0 | 3.8 | | 562 | | 0 | 2.5 | | 653 | | 0 | 5.5 | 651 | | | 0 | 4.8 | |  |
|  |  |  |  |  | |  |  | |  | | |  |  | |  | |  |  | |  | |  |  |  | | |  |  | |  |
| ***Non-ST-segment elevation acute coronary syndrome (NSTEACS):*** | | | | | | | | | | | | | | | | | | | | | | | | | | | | |  |  |
| Aspirin & heparin | 7, 598 | *0* | 91.6 | 1,220 | | 0 | 99.9 | | 1,368 | | | 0 | 99.9 | | 1,508 | | 0 | 88.6 | | 1,652 | | 0 | 81.5 | 1,852 | | | 0 | 91.7 | |  |
| Aspirin alone | 7, 598 | *12.4* | 8.4 | 1,220 | | 12.4 | 0.1 | | 1,368 | | | 12.4 | 0.1 | | 1,508 | | 12.4 | 11.4 | | 1,652 | | 12.4 | 18.5 | 1,852 | | | 12.4 | 8.3 | |  |
| Platelet glycoprotein IIB/IIIA inhibitors | 7, 598 | *0* | 12.8 | 1,220 | | 0 | 12.8 | | 1,368 | | | 0 | 12.7 | | 1,508 | | 0 | 12.7 | | 1,652 | | 0 | 12.9 | 1,852 | | | 0 | 12.9 | |  |
| ACE I/ARB | 7, 598 | *0* | 56.2 | 1,220 | | 0 | 55.5 | | 1,368 | | | 0 | 57.4 | | 1,508 | | 0 | 57.7 | | 1,652 | | 0 | 56.7 | 1,852 | | | 0 | 53.9 | |  |
| B-Blocker | 7, 598 | *14.8* | 85.5 | 1,220 | | 14.3 | 78.2 | | 1,368 | | | 14.8 | 86.7 | | 1,508 | | 14.6 | 90.3 | | 1,652 | | 15.2 | 88.8 | 1,852 | | | 15.1 | 82.7 | |  |
| Clopidogrel | 7, 598 | *0* | 95.7 | 1,220 | | 0 | 98.0 | | 1,368 | | | 0 | 96.5 | | 1,508 | | 0 | 95.0 | | 1,652 | | 0 | 94.7 | 1,852 | | | 0 | 94.9 | |  |
| CABG (< 6 weeks) | 7, 598 | *4.3* | 6.3 | 1,220 | | 4.5 | 6.7 | | 1,368 | | | 6.8 | 6.0 | | 1,508 | | 4.0 | 7.2 | | 1,652 | | 3.1 | 6.1 | 1,852 | | | 3.6 | 5.8 | |  |
| PCI (0-14 days) | 7, 598 | *0* | 26.3 | 1,220 | | 0 | 26.7 | | 1,368 | | | 0 | 25.8 | | 1,508 | | 0 | 26.7 | | 1,652 | | 0 | 26.6 | 1,852 | | | 0 | 25.7 | |  |
| ***Secondary prevention post myocardial infarction:*** | | | | | | | | | | | | | | | | | | | | | | | | | | | | |  |  |
| Aspirin | 57,198 | *6.0* | 72.4 | 8,543 | | 6.0 | 68.0 | | 10,312 | | | 6.0 | 73.3 | | 11,624 | | 6.0 | 73.8 | | 12,921 | | 6.0 | 74.6 | 13,798 | | | 6.0 | 71.3 | |  |
| B-Blocker | 57,198 | *12.2* | 53.7 | 8,543 | | 12.1 | 48.7 | | 10,312 | | | 12.1 | 53.6 | | 11,624 | | 12.2 | 53.9 | | 12,921 | | 12.3 | 56.5 | 13,798 | | | 12.3 | 54.0 | |  |
| ACE I/ARB | 57,198 | *0* | 41.9 | 8,543 | | 0 | 41.5 | | 10,312 | | | 0 | 42.3 | | 11,624 | | 0 | 42.4 | | 12,921 | | 0 | 42.4 | 13,798 | | | 0 | 41.0 | |  |
| Statin | 57,198 | *0.4* | 68.0 | 8,543 | | 0.4 | 66.6 | | 10,312 | | | 0.4 | 69.4 | | 11,624 | | 0.4 | 69.0 | | 12,921 | | 0.4 | 69.6 | 13,798 | | | 0.4 | 65.4 | |  |
| Warfarin | 57,198 | *2.0* | 1.7 | 8,543 | | 2.0 | 1.9 | | 10,312 | | | 2.0 | 1.8 | | 11,624 | | 2.0 | 1.6 | | 12,921 | | 2.0 | 1.6 | 13,798 | | | 2.0 | 1.5 | |  |
| ***Secondary prevention post revascularisation:*** | | | | | | | | | | | | | | | | | | | | | | | | | | | | |  |  |
| Aspirin | 15,593 | *5.6* | 81.8 | 2,463 | | 5.8 | 80.1 | | 2,929 | | | 5.7 | 81.4 | | 3,268 | | 5.5 | 82.9 | | 3,517 | | 5.4 | 81.5 | 3,416 | | | 5.5 | 82.6 | |  |
| B-Blocker | 15,593 | *16.3* | 52.8 | 2,463 | | 16.0 | 48.8 | | 2,929 | | | 16.2 | 51.5 | | 3,268 | | 16.4 | 53.1 | | 3,517 | | 16.5 | 53.7 | 3,416 | | | 16.4 | 55.9 | |  |
| ACE I/ARB | 15,593 | *0* | 42.8 | 2,463 | | 0 | 43.4 | | 2,929 | | | 0 | 41.0 | | 3,268 | | 0 | 42.4 | | 3,517 | | 0 | 43.7 | 3,416 | | | 0 | 43.4 | |  |
| Statin | 15,593 | *0.4* | 80.7 | 2,463 | | 0.4 | 82.1 | | 2,929 | | | 0.4 | 80.4 | | 3,268 | | 0.4 | 80.9 | | 3,517 | | 0.4 | 80.7 | 3,416 | | | 0.4 | 79.6 | |  |
| Warfarin | 15,593 | *10.5* | 1.2 | 2,463 | | 11.0 | 1.3 | | 2,929 | | | 10.7 | 0.9 | | 3,268 | | 10.4 | 1.3 | | 3,517 | | 10.0 | 1.3 | 3,416 | | | 10.3 | 1.2 | |  |

| ***Chronic stable coronary artery disease:*** | | | | | | | | | | | | | | | | | | | | |
| --- | --- | --- | --- | --- | --- | --- | --- | --- | --- | --- | --- | --- | --- | --- | --- | --- | --- | --- | --- | --- |
| Aspirin | 71,176 | | 5.6 | | 45.9 | 11,387 | 5.6 | 39.9 | 12,923 | 5.6 | 44.7 | 14,271 | 5.6 | 46.5 | 15,565 | 5.6 | 49.0 | 17,030 | 5.6 | 47.3 |
| Statins | 71,176 | | 0.4 | | 38.9 | 11,387 | 0.4 | 36.2 | 12,923 | 0.4 | 39.7 | 14,271 | 0.4 | 39.5 | 15,565 | 0.4 | 40.3 | 17,030 | 0.4 | 38.4 |
| ACE I/ARB | 71,176 | | 0 | | 31.8 | 11,387 | 0 | 29.6 | 12,923 | 0 | 32.0 | 14,271 | 0 | 32.3 | 15,565 | 0 | 32.7 | 17,030 | 0 | 31.7 |
| CABG | 71,176 | | 1.4 | | 1.1 | 11,387 | 2.2 | 1.1 | 12,923 | 1.1 | 1.3 | 14,271 | 1.1 | 1.1 | 15,565 | 1.4 | 1.2 | 17,030 | 1.0 | 1.0 |
| **Heart failure in patients requiring hospitalisation:** | | | | | | | | | | | | | | | | | | | | |
| ACE I/ARB | 6,317 | 0 | | | 56.3 | 721 | 0 | 55.2 | 987 | 0 | 56.1 | 1,276 | 0 | 55.7 | 1,532 | 0 | 57.1 | 1,802 | 0 | 56.5 |
| B-Blocker | 6,317 | 5.1 | | | 49.1 | 721 | 5.0 | 49.0 | 987 | 5.0 | 48.4 | 1,276 | 5.1 | 48.2 | 1,532 | 5.1 | 49.4 | 1,802 | 5.1 | 49.8 |
| Spironolactone | 6,317 | 0 | | | 27.6 | 721 | 0 | 22.2 | 987 | 0 | 24.9 | 1,276 | 0 | 28.5 | 1,532 | 0 | 28.7 | 1,802 | 0 | 29.7 |
| Aspirin | 6,317 | 8.9 | | | 51.8 | 721 | 8.8 | 45.8 | 987 | 8.7 | 51.2 | 1,276 | 8.8 | 52.3 | 1,532 | 9.0 | 53.0 | 1,802 | 9.1 | 53.0 |
| **Heart failure in the community:** | | | | | | | | | | | | | | | | | | | | |
| ACE I/ARB | 22,856 | | | 0 | 54.7 | 3,034 | 0 | 55.0 | 3,833 | 0 | 54.1 | 4,590 | 0 | 54.5 | 5,411 | 0 | 54.5 | 5,989 | 0 | 55.2 |
| B-Blocker | 22,856 | | | 5.5 | 45.1 | 3,034 | 5.3 | 45.9 | 3,833 | 5.4 | 44.4 | 4,590 | 5.5 | 43.9 | 5,411 | 5.6 | 45.4 | 5,989 | 5.5 | 45.7 |
| Spironolactone | 22,856 | | | 0 | 18.2 | 3,034 | 0 | 14.6 | 3,833 | 0 | 16.5 | 4,590 | 0 | 18.2 | 5,411 | 0 | 19.2 | 5,989 | 0 | 20.1 |
| Aspirin | 22,856 | | | 5.3 | 51.7 | 3,034 | 5.2 | 46.8 | 3,833 | 5.3 | 51.7 | 4,590 | 5.3 | 52.0 | 5,411 | 5.3 | 52.4 | 5,989 | 5.3 | 53.3 |
| ***Numbers with CHD*** | 183,570 | | |  |  | 27,801 |  |  | 32,884 |  |  | 37,098 |  |  | 41,250 |  |  | 44,536 |  |  |
|  | | | | | | | | | | | | | | | | | | | | |
| ***Primary prevention therapies:*** | | | | | | | | | | | | | | | | | | | | |
| Statins | 1,836,236 | | 0.4 | | 14.7 | 351,550 | 0.3 | 14.3 | 356,332 | 0.3 | 14.9 | 380,546 | 0.3 | 14.4 | 391,676 | 0.3 | 14.6 | 356,131 | 0.4 | 15.4 |
| Anti-hypertension | 1,114,607 | | *11.7* | | 32.0 | 200,908 | 11.0 | 32.1 | 228,265 | 11.7 | 30.6 | 231,861 | 12.6 | 31.3 | 232,008 | 12.5 | 33.2 | 221,565 | 10.6 | 32.8 |

Abbreviations: ACE I, angiotensin converting enzyme inhibitor; ARB, angiotensin receptor blocker; B-blocker, beta-blocker; CABG, coronary artery bypass graft; PCI, percutaneous coronary intervention; SECQ, socio-economic quintile

# Table S8. Beta coefficients for major risk factors

**Estimated β coefficients from multiple regression analyses for the relationship between absolute changes in population mean risk factors and percentage changes in coronary heart disease mortality for men and women, stratified by age. Data sources, values and comments.**

| **Systolic blood pressure** | | **Age group (years)** | | | | |
| --- | --- | --- | --- | --- | --- | --- |
|  | | **25-44** | **45-54** | **55-64** | **65-74** | **75+** |
|  | |  |  |  |  |  |
| **Men** (hazard ratio per 20 mmHg) | | 0.49 | 0.49 | 0.52 | 0.58 | 0.65 |
| Men (log hazard ratio per 1 mmHg) | | **-0.036** | **-0.035** | **-0.032** | **-0.027** | **-0.021** |
|  | |  |  |  |  |  |
| *Minimum* | | *-0.029* | *-0.028* | *-0.026* | *-0.022* | *-0.017* |
| *Maximum* | | *-0.043* | *-0.042* | *-0.039* | *-0.032* | *-0.025* |
|  | |  |  |  |  |  |
|  | |  |  |  |  |  |
| **Women** (hazard ratio per 20 mmHg) | | 0.40 | 0.40 | 0.49 | 0.52 | 0.59 |
| Women (log hazard ratio per 1 mmHg) | | **-0.046** | **-0.046** | **-0.035** | **-0.032** | **-0.026** |
| *Minimum* | | *-0.037* | *-0.037* | *-0.028* | *-0.026* | *-0.021* |
| *Maximum* | | *-0.055* | *-0.055* | *-0.042* | *-0.039* | *-0.031* |
|  | |  |  |  |  |  |
|  | |  |  |  |  |  |
| Source: Prospective studies collaborative meta-analysis, Lancet 2002(49) | | | | | | |
| Units: Percentage change in CHD mortality per 20 mmHg change in systolic blood pressure | | | | | | |
| **Strengths:** | Large dataset, includes US data, adjusted for regression dilution bias, consistent with randomised controlled trials, results stratified by age and sex, with 95% confidence intervals | | | | | |
| **Limitations:** | Some publication bias still possible | | | | | |

| **Cholesterol** | **Age groups (years)** | | | | | | |
| --- | --- | --- | --- | --- | --- | --- | --- |
|  | **25-44** | | **45-54** | **55-64** | **65-74** | **75-84** | **85+** |
| **Mortality reduction per 1 mmol/l** | | | | | | | |
| Men | 0.55 | | 0.53 | 0.36 | 0.21 | 0.21 | 0.21 |
| Women | 0.57 | | 0.52 | 0.35 | 0.23 | 0.23 | 0.23 |
| **Log coefficient** | | | | | | | |
| **Men** | **-0.799** | | **-0.755** | **-0.446** | **-0.236** | **-0.117** | **-0.083** |
| *Minimum* | *-0.639* | | *-0.604* | *-0.357* | *-0.189* | *-0.093* | *-0.067* |
| *Maximum* | *-0.958* | | *-0.906* | *-0.536* | *-0.283* | *-0.140* | *-0.100* |
|  |  | |  |  |  |  |  |
| **Women** | **-0.844** | | **-0.734** | **-0.431** | **-0.261** | **-0.174** | **-0.051** |
| *Minimum* | *-0.675* | | *-0.587* | *-0.345* | *-0.209* | *-0.139* | *-0.041* |
| *Maximum* | *-1.013* | | *-0.881* | *-0.517* | *-0.314* | *-0.209* | *-0.062* |
| Source: Prospective studies collaborative meta-analysis, Lancet 2007(9) | | | | | | | |
| Units: | | Percentage change in CHD mortality per 1 mmol/l change in total cholesterol | | | | | |
| **Strengths:** | | Includes US data, adjusted for regression dilution bias, includes randomised controlled trials, RCT values consistent with observational data, results stratified by age and sex, with 95% confidence intervals | | | | | |
| **Limitations:** | | Some publication bias still possible | | | | | |

| **Body Mass Index (BMI)** | | **Age groups (years)** | | | | |
| --- | --- | --- | --- | --- | --- | --- |
|  | | **<44** | **45-59** | **60-69** | **70-79** | **80+** |
| *James et.al (2004):* | |  |  |  |  |  |
| Hazard ratio | | 0.89 | 0.91 | 0.95 | 0.96 | 0.97 |
| Risk reduction† per 1 kg/m^2^ | | 0.11 | 0.09 | 0.05 | 0.04 | 0.03 |
| Age gradient (45-59 as reference) | | 1.22 | **1.00** | 0.56 | 0.44 | 0.33 |
| *Bogers (2006):*  Relative risks, CHD deaths per 5 BMI units (kg/m^2^) | |  | **1.16** |  |  |  |
| Relative risks per 1 kg/m^2^ applying age gradients from James et.al | | 1.04 | 1.03 | 1.02 | 1.01 | 1.01 |
| **Log coefficients** | | **0.0363** | **0.0297** | **0.0165** | **0.0132** | **0.0099** |
| *Minimum* | | *0.0255* | *0.0209* | *0.0116* | *0.0093* | *0.0070* |
| *Maximum* | | *0.0466* | *0.0381* | *0.0212* | *0.0169* | *0.0127* |
| Source: Bogers et al (2007) and James et al (2004)(50,51) | | | | | | |
| Units: | Percentage change in CHD mortality per 1 kg/m^2^ change in BMI | | | | | |
| **Strengths:** | Large number of studies included. Adjusted for blood pressure, total cholesterol, and physical activity. 95% confidence intervals included. | | | | | |
| **Limitations:** | Observational data; age gradient applied from James study | | | | | |

^†^ Risk reduction = 1 – hazard ratio

# Table S9. Relative Risks for CHD used in the IMPACT_SEC_ model for Smoking, Diabetes and Physical Inactivity

**Calculation of Relative Risk estimates for dichotomous risk factors in the IMPACT_SEC_ model**

Relative risks (RRs) estimated by expert working groups for the World Health Organization’s (WHO) Global Burden of Disease (GBD) 2001 Study were used for smoking and physical activity.(11) Effect estimates were based on systematic reviews of cohort studies (adjusted for regression dilution bias) and meta-analyses of RCTs. Age-variation in the relative risks for diabetes were taken from the DECODE study.(12) These were then applied to the gender-variation in relative risks estimated by Huxley et al.(13) The set of RRs used in the IMPACT_SEC_ model for the three binary risk factors with 95% Confidence Intervals (in parentheses) are shown below. The RRs were assumed constant across deprivation quintiles.

|  | **Smoking** | **Physical inactivity** | **Diabetes** |
| --- | --- | --- | --- |
| **Male 25-34** | 5.51 (2.47-12.25) | 1.50 (1.35-1.67) | 4.33 (3.47-5.20) |
| **Male 35-44** | 5.51 (2.47-12.25) | 1.50 (1.35-1.67) | 3.22 (2.58-3.86) |
| **Male 45-54** | 3.04 (2.66-3.48) | 1.50 (1.35-1.67) | 2.14 (1.71-2.57) |
| **Male 55-64** | 2.51 (2.22-2.84) | 1.50 (1.35-1.67) | 1.99 (1.59-2.39) |
| **Male 65-74** | 1.69 (1.52-1.89) | 1.44 (1.30-1.61) | 1.86 (1.49-2.23) |
| **Male 75-84** | 1.31 (1.11-1.56) | 1.32 (1.19-1.47) | 1.71 (1.37-2.05) |
| **Male 85+** | 1.05 (0.78-1.43) | 1.23 (1.11-1.37) | 1.71 (1.37-2.05) |
| **Female 25-34** | 2.26 (0.83-6.14) | 1.50 (1.35-1.68) | 7.55 (6.04-9.06) |
| **Female 35-44** | 2.26 (0.83-6.14) | 1.50 (1.35-1.68) | 5.63 (4.51-6.76) |
| **Female 45-54** | 3.78 (3.10-4.62) | 1.50 (1.35-1.68) | 3.81 (3.05-4.57) |
| **Female 55-64** | 3.21 (2.70-3.82) | 1.50 (1.35-1.68) | 3.12 (2.50-3.74) |
| **Female 65-74** | 2.17 (1.89-2.47) | 1.45 (1.30-1.61) | 2.55 (2.04-3.06) |
| **Female 75-84** | 1.58 (1.33-1.88) | 1.33 (1.20-1.47) | 2.36 (1.89-2.83) |
| **Female 85+** | 1.38 (1.08-1.77) | 1.24 (1.13-1.37) | 2.36 (1.89-2.83) |

In Section S9.1 we list the published RRs for each of the three risk factors; in Section S9.2 we detail how these were modified to fit to the age-gender distributions used in the IMPACT_SEC_ model.

S9.1 Published relative risks

**1 Current smoking**

Relative risk of mortality from Ischaemic Heart Disease (ICD9: 410-414) for current smokers relative to non-smokers (95% CIs in parentheses), from the American Cancer Society’s Cancer Prevention Study (CPS-II)

| **Age** | **Male** | **Female** |
| --- | --- | --- |
| **30-44** | 5.51 (2.47-12.25) | 2.26 (0.83-6.14) |
| **45-59** | 3.04 (2.66-3.48) | 3.78 (3.10-4.62) |
| **60-69** | 1.88 (1.70-2.08) | 2.53 (2.22-2.87) |
| **70-79** | 1.44 (1.27-1.63) | 1.68 (1.46-1.93) |
| **≥ 80 years** | 1.05 (0.78-1.43) | 1.38 (1.08-1.77) |

Notes: CPS-II is an ongoing prospective study of mortality in 1.2 million Americans aged 30 years or more when they completed a questionnaire on tobacco and alcohol use, diet, and multiple other factors affecting health and mortality in 1982. RRs were estimated from Cox proportional-hazard models, with non-smokers as the reference group (RR=1.0 for non-smokers). Risks were adjusted for age, race, education, marital status, “blue collar” employment in most recent or current job, weekly consumption of vegetables and citrus fruit, vitamin (A, C, and E) use, alcohol use, aspirin use, body mass index, exercise, dietary fat consumption and for hypertension and diabetes (both at baseline). Analyses of the hazards associated with smoking were based on the first six years of follow-up (1982 through 1988).

*Source: Ezzati et al (2005)(52)*

**2 Physical inactivity**

Relative risk of Ischaemic Heart Disease (ICD10: I20-I25) from physical (in)activity levels from WHO GBD Study (95% CIs in parentheses), relative to those considered physically active

| **Age** | **Inactive level** | **Insufficiently active level** |
| --- | --- | --- |
| **15-69** | 1.71 (1.58-1.85) | 1.44 (1.28-1.62) |
| **70-79** | 1.50 (1.38-1.61) | 1.31 (1.17-1.48) |
| **80+ years** | 1.30 (1.21-1.41) | 1.20 (1.07-1.35) |

*Notes*: Physical (in)activity in the WHO GBD study was treated as a categorical variable with three categories: Level 1: Inactive: ‘doing no or very little physical activity at work, at home, for transport, or during discretionary time’. Level 2: Insufficiently active: ‘doing some physical activity but less than 150 minutes of moderate-intensity physical activity or 60 minutes of vigorous-intensity physical activity a week accumulated across work, home, transport or discretionary domains’. Level 3: Sufficiently active (unexposed): ‘at least 150 minutes of moderate-intensity physical activity or 60 minutes of vigorous-intensity physical activity a week accumulated across work, home, transport or discretionary domains’, which approximately corresponds to current recommendations in many countries. RR estimates were adjusted for confounding variables, measurement error associated with self-report, and attenuated over age (25% of the excess risk for the 70-79 year age-group and 50% of the excess risk for the oldest age group, 80+), but not adjusted for blood pressure and cholesterol.

*Sources: Bull et al (2004)and Joubert et al (2007)* (53,54)

**3 Diabetes mellitus**

A meta-analysis of 22 prospective cohort studies by Huxley et al11 estimated that the relative risk for CHD due to diabetes was 1.99 (95% CI: 1.69-2.35) in men and 3.12 (2.34-4.17) in women. These estimates were derived from studies that provided multiple risk factor adjusted coefficients. This systematic review included Asia-Pacific studies with larger RR values compared to Western studies, although the difference was not statistically significant. To obtain age-specific relative risk estimates, we used the age-gradients in relative risk for total mortality for diabetic persons compared to non-diabetics taken from the DECODE study as detailed below.

**Estimates of relative risk for total mortality due to diabetes (DECODE study)**

| **Age** | **Males** | **Females** |
| --- | --- | --- |
| **20-29** | 3.66 | 6.05 |
| **30-39** | 3.38 | 5.41 |
| **40-49** | 1.85 | 3.14 |
| **50-59** | 1.63 | 2.64 |
| **60-69** | 1.60 | 2.04 |
| **70-79** | 1.39 | 1.79 |

*Notes*: Undertaken in 1997, the Diabetes Epidemiology: Collaborative Analysis of Diagnostic Criteria in Europe (DECODE) study built a dataset that included the baseline values needed to determine the presence of the metabolic syndrome using a modification of the WHO definition for 11 European study cohorts, and follow-up data on all-cause and cardiovascular disease mortality.(55)

*Source: Roglic and Unwin (2010)* (12)

S9.2 **Adjusting the published RR values**

The published relative risk values were adjusted to conform to the age distributions and binary classification of risk used in the IMPACTSEC study. Table S9 above shows the final, adjusted RR values used in our model to estimate the Population Attributable Risk Fractions. Below we detail how the adjustments to the published RR values were calculated.

*Weighted averages using the European Standard Population*

We adjusted the RRs for each binary risk factor to match the 10 year age-bands used in our study. A population-weighted approach, using weights from the European Union (EU) reference population, was used to estimate the RRs for each of the 7 age-bands. We used the EU standard reference population for two reasons: first, for consistency. The EU standard was used as the reference population distribution in all IMPACTSEC and related studies to calculate directly-standardised rates. Secondly, to aid comparability. The ensuing age-weighted rates can easily be used in studies in other European countries with a similar population structure and results compared against each other or with other health statistics (e.g. mortality rates) standardized using the same reference population.

**1 Adjustment to published RRs for current smoking**

The population-weighted adjustment approach is illustrated below using the RRs for current smoking in men as an example. For example, the RR value used in our model for males aged 55-64 was 2.51. This is roughly, but not exactly, halfway between the CPS-II estimates of 3.04 and 1.88 for males aged 55-59 and 60-64 with population weights of 0.545 and 0.455, respectively.

| **Age bands for IMPACTSEC** | **5 year age-bands** | **EU population** | **EU population weight** | **CPS-II RR** | **IMPACTSEC RR** |
| --- | --- | --- | --- | --- | --- |
| **M 25-34** | **M 25-29** | 7000 | 0.5 | 5.51 | **5.51** |
|  | **M 30-34** | 7000 | 0.5 | 5.51 |  |
| **M 35-44** | **M 35-39** | 7000 | 0.5 | 5.51 | **5.51** |
|  | **M 40-44** | 7000 | 0.5 | 5.51 |  |
| **M 45-54** | **M 45-49** | 7000 | 0.5 | 3.04 | **3.04** |
|  | **M 50-54** | 7000 | 0.5 | 3.04 |  |
| **M 55-64** | **M 55-59** | 6000 | 0.545 | 3.04 | **2.51** |
|  | **M 60-64** | 5000 | 0.455 | 1.88 |  |
| **M 65-74** | **M 65-69** | 4000 | 0.571 | 1.88 | **1.69** |
|  | **M 70-74** | 3000 | 0.429 | 1.44 |  |
| **M 75-84** | **M 75-79** | 2000 | 0.667 | 1.44 | **1.31** |
|  | **M 80-84** | 1000 | 0.333 | 1.05 |  |
| **M 85+** | | 1000 | 1 | 1.05 | **1.05** |

**2 Adjustment to published RRs for physical activity**

We adjusted the published RRs for physical activity to employ a dichotomous rather than trichotomous measure (i.e. combining the GBD ‘insufficiently active’ and ‘inactive’ categories into a single inactive group). We used a weighted average approach using as weights the GBD estimates of exposure to physical inactivity in the EUR-A subregion (Table 10.10 in Bull et al, 2004).(53) The physical activity exposure levels (%) with the corresponding RR value (RR=1 for the Level 3 ‘sufficiently active’ group) shown in parentheses are detailed below.

**Physical activity exposure levels (%) in the GBD EUR-A region† with accompanying RRs**

| **Exposure category** | **Age-group (years)** | | | | | |
| --- | --- | --- | --- | --- | --- | --- |
|  | 15-29 | 30-44 | 45-59 | 60-69 | 70-79 | ≥ 80 years |
| **Men:** |  |  |  |  |  |  |
| **Level 3: Recommended** | 35 (1) | 29 (1) | 30 (1) | 30 (1) | 30 (1) | 32 (1) |
| **Level 2: Insufficient** | 52 (1.44) | 57 (1.44) | 55 (1.44) | 52 (1.44) | 50 (1.31) | 47 (1.20) |
| **Level 1: Inactive** | 13 (1.71) | 15 (1.71) | 16 (1.71) | 18 (1.71) | 20 (1.50) | 21 (1.30) |
| **Levels 1 and 2**  **(combined RR)** | 1.49 | 1.50 | 1.50 | 1.51 | 1.36 | 1.23 |
|  |  |  |  |  |  |  |
| **Women:** |  |  |  |  |  |  |
| **Level 3: Recommended** | 37 (1) | 31 (1) | 31 (1) | 33 (1) | 31 (1) | 30 (1) |
| **Level 2: Insufficient** | 47 (1.44) | 51 (1.44) | 51 (1.44) | 45 (1.44) | 45 (1.31) | 42 (1.20) |
| **Level 1: Inactive** | 17 (1.71) | 18 (1.71) | 18 (1.71) | 22 (1.71) | 24 (1.50) | 28 (1.30) |
| **Levels 1 and 2**  **(combined RR)** | 1.51 | 1.51 | 1.51 | 1.53 | 1.38 | 1.24 |

*Notes*: For example, using the GBD estimates of exposure and RRs, the combined RR for males aged 45-59 equalled 1.50:

RR (combining insufficient and inactive): ((0.55×1.44)+(0.16×1.71))/(0.55+0.16) = 1.50

† Countries in the EUR-A GBD subregion: Andorra, Austria, Belgium, Croatia, Czech Republic, Denmark, Finland, France, Germany, Greece, Iceland, Ireland, Israel, Italy, Luxembourg, Malta, Monaco, Netherlands, Norway, Portugal, San Marino, Slovenia, Spain, Sweden, Switzerland, United Kingdom

*Sources: Bull et al (2004)* (53)

Due to negligible differences in the combined RRs across the youngest age categories we used a relative risk of 1.50 for men and women aged 25-69. Having used the GBD data to obtain a set of RRs for a binary physical activity variable in each of three broad age-groups (25-69, 70-79, 80+) we then used the population-weighted average approach, using weights from the EU population, to estimate the combined RRs for the seven 10-year age bands used in IMPACTSEC. This is illustrated below for males.

| **Age bands for IMPACTSEC** | **5 year age-bands** | **EU population** | **EU population weight** | **Combined inactive RR** | **IMPACTSEC RR** |
| --- | --- | --- | --- | --- | --- |
| **M 25-34** | **M 25-29** | 7000 | 0.5 | 1.50 | 1.50 |
|  | **M 30-34** | 7000 | 0.5 | 1.50 |  |
| **M 35-44** | **M 35-39** | 7000 | 0.5 | 1.50 | 1.50 |
|  | **M 40-44** | 7000 | 0.5 | 1.50 |  |
| **M 45-54** | **M 45-49** | 7000 | 0.5 | 1.50 | 1.50 |
|  | **M 50-54** | 7000 | 0.5 | 1.50 |  |
| **M 55-64** | **M 55-59** | 6000 | 0.545 | 1.50 | 1.50 |
|  | **M 60-64** | 5000 | 0.455 | 1.50 |  |
| **M 65-74** | **M 65-69** | 4000 | 0.571 | 1.50 | 1.44 |
|  | **M 70-74** | 3000 | 0.429 | 1.36 |  |
| **M 75-84** | **M 75-79** | 2000 | 0.667 | 1.36 | 1.32 |
|  | **M 80-84** | 1000 | 0.333 | 1.23 |  |
| **M 85+** | | 1000 | 1 | 1.23 | 1.23 |

**3 Adjustment to published RRs for diabetes**

Unlike the relative risk estimates for smoking and physical activity, the meta-analysis of 22 prospective cohort studies by Huxley et al(13) provided just overall estimates of RR for diabetes by gender. From previous studies we know that the relative risks associated with diabetes are higher for women and decline with age. To obtain age variation in the diabetes RRs, we used the age-gradient in the RR estimates for total mortality taken from the DECODE study.(12) As diabetes is a proximate risk factor for cardiovascular disease CVD) and CVD comprises about half of total mortality(56), we have assumed that the relative age pattern of diabetes-related CHD mortality will be similar. We made the reasonable assumption that the mean age across the prospective studies examined in the meta-analysis by Huxley et al(13) was age 55-64 years. Hence the age-adjusted values were anchored to this age group for both sexes.

To estimate the relative risks for diabetes, we first used the DECODE study estimates to compute the age gradient in RRs, indexed on the value for the 55-64 age-group. The resulting value for each age group was then multiplied by the overall RR for men and women (1.99 and 3.12, respectively) taken from the study by Huxley et al(13) to give the age-specific RR of CHD mortality for diabetes. For ages 80 and over (for which published RRs were not found), we have assumed that the RR remained the same as for those aged 70-79. Detailed below is the worked example for males.

| **Age-bands for IMPACTSEC** | **5 year age bands** | **EU pop** | **EU pop weight** | **RRa from DECODE study** | **Weighted RRs from DECODE** | **Age variation from DECODE†** | **IMPACTSEC RRs‡** |
| --- | --- | --- | --- | --- | --- | --- | --- |
| **M 25-34** | **M 25-29** | 7000 | 0.5 | 3.66 | 3.5 | 2.18 | 4.33 |
|  | **M 30-34** | 7000 | 0.5 | 3.38 |  |  |  |
| **M 35-44** | **M 35-39** | 7000 | 0.5 | 3.38 | 2.6 | 1.62 | 3.22 |
|  | **M 40-44** | 7000 | 0.5 | 1.85 |  |  |  |
| **M 45-54** | **M 45-49** | 7000 | 0.5 | 1.85 | 1.7 | 1.08 | 2.14 |
|  | **M 50-54** | 7000 | 0.5 | 1.63 |  |  |  |
| **M 55-64** | **M 55-59** | 6000 | 0.545 | 1.63 | 1.6 | **1.00** | **1.99** |
|  | **M 60-64** | 5000 | 0.455 | 1.60 |  |  |  |
| **M 65-74** | **M 65-69** | 4000 | 0.571 | 1.60 | 1.5 | 0.93 | 1.86 |
|  | **M 70-74** | 3000 | 0.429 | 1.39 |  |  |  |
| **M 75-84** | **M 75-79** | 2000 | 0.667 | 1.39 | 1.4 | 0.86 | 1.71 |
|  | **M 80-84** | 1000 | 0.333 | 1.39 |  |  |  |
| **M 85+** |  | 1000 | 1 | 1.39 | 1.4 | 0.86 | 1.71 |

*Notes:*

a RR for overall mortality

† 55-64 age-group taken as the reference

‡ IMPACTSEC RR (for CHD mortality in DM) calculated as sex-specific RR from Huxley et al (2006)(13) (men:1.99; women:3.12) multiplied by age-variation in EU weighted RRs taken from DECODE study(12)

#

# Table S10. Risk factor levels in 1991 and 2007 by sex and socioeconomic quintiles

# (Data from Copenhagen City Heart Study 3 and 4)*

|  | **Denmark** | | **SECQ1** | | **SECQ2** | | **SECQ3** | | **SECQ4** | | **SECQ5** | |
| --- | --- | --- | --- | --- | --- | --- | --- | --- | --- | --- | --- | --- |
|  | **1991** | **2007** | **1991** | **2007** | **1991** | **2007** | **1991** | **2007** | **1991** | **2007** | **1991** | **2007** |
| **Smoking prevalence (%)** |  |  |  |  |  |  |  |  |  |  |  |  |
| Male | 55.7 | 28.1 | 44.8 | 19.2 | 53.9 | 24.5 | 60.4 | 29.8 | 63.0 | 34.3 | 61.3 | 37.5 |
| Female | 49.4 | 28.2 | 39.5 | 16.3 | 47.5 | 23.6 | 52.5 | 29.9 | 53.4 | 33.6 | 49.9 | 33.6 |
| **Diabetes prevalence (%)** |  |  |  |  |  |  |  |  |  |  |  |  |
| Male | 1.6 | 2.6 | 1.2 | 1.5 | 1.5 | 1.9 | 1.7 | 2.5 | 1.9 | 3.3 | 2.0 | 4.0 |
| Female | 1.5 | 2.5 | 0.9 | 0.9 | 1.3 | 1.5 | 1.5 | 1.9 | 1.7 | 2.4 | 2.0 | 2.9 |
| **Physical inactivity (%)** |  |  |  |  |  |  |  |  |  |  |  |  |
| Male | 12.6 | 8.0 | 9.2 | 6.1 | 12.2 | 6.6 | 14.8 | 7.7 | 16.1 | 9.8 | 15.8 | 13.2 |
| Female | 12.0 | 7.1 | 10.0 | 5.9 | 12.0 | 7.0 | 14.0 | 7.9 | 15.8 | 8.3 | 17.2 | 8.2 |
| **Systolic blood pressure, mmHg** | | |  |  |  |  |  |  |  |  |  |  |
| Male | 137.2 | 132.6 | 136.5 | 131.9 | 136.1 | 134.0 | 139.0 | 129.9 | 136.9 | 134.0 | 137.6 | 133.1 |
| Female | 131.7 | 128.5 | 130.6 | 127.0 | 131.3 | 128.3 | 131.3 | 131.1 | 131.9 | 128.9 | 133.4 | 127.1 |
| **Cholesterol, mmol/L** |  |  |  |  |  |  |  |  |  |  |  |  |
| Male | 6.0 | 5.1 | 6.0 | 5.1 | 6.0 | 5.1 | 6.1 | 5.1 | 6.0 | 5.2 | 5.9 | 5.1 |
| Female | 6.1 | 5.1 | 6.0 | 5.1 | 6.1 | 5.1 | 6.2 | 5.1 | 6.2 | 5.0 | 6.1 | 5.3 |
| **Body mass index, kg/m^2^** |  |  |  |  |  |  |  |  |  |  |  |  |
| Male | 25.8 | 26.3 | 25.4 | 25.7 | 25.7 | 26.2 | 26.3 | 26.6 | 26.2 | 26.5 | 25.7 | 26.6 |
| Female | 24.8 | 25.1 | 24.2 | 24.4 | 24.5 | 24.8 | 25.1 | 25.6 | 25.1 | 25.2 | 25.1 | 25.7 |

***** Diabetes data are from the Danish National Patients Registry

# Table S10a. Risk factor levels in 1991 and 2007 by sex and socioeconomic quintiles (Data from MONICA III and Health 2006)*

|  | **Denmark** | | **SECQ1** | | **SECQ2** | | **SECQ3** | | **SECQ4** | | **SECQ5** | |
| --- | --- | --- | --- | --- | --- | --- | --- | --- | --- | --- | --- | --- |
|  | **1991** | **2007** | **1991** | **2007** | **1991** | **2007** | **1991** | **2007** | **1991** | **2007** | **1991** | **2007** |
| **Smoking prevalence (%)** |  |  |  |  |  |  |  |  |  |  |  |  |
| Male | 52.3 | 7.8 | 44.5 | 5.9 | 52.4 | 8.0 | 46.7 | 9.5 | 56.6 | 9.1 | 60.7 | 6.4 |
| Female | 44.5 | 12.3 | 35.7 | 8.7 | 44.5 | 11.0 | 53.0 | 13.3 | 50.1 | 14.7 | 42.8 | 13.9 |
| **Diabetes prevalence (%)** |  |  |  |  |  |  |  |  |  |  |  |  |
| Male | 1.6 | 2.6 | 1.2 | 1.5 | 1.5 | 1.9 | 1.7 | 2.5 | 1.9 | 3.3 | 2.0 | 4.0 |
| Female | 1.5 | 2.5 | 0.9 | 0.9 | 1.3 | 1.5 | 1.5 | 1.9 | 1.7 | 2.4 | 2.0 | 2.9 |
| **Physical inactivity (%)** |  |  |  |  |  |  |  |  |  |  |  |  |
| Male | 25.5 | 7.3 | 18.2 | 8.4 | 18.6 | 8.1 | 22.0 | 7.8 | 29.7 | 6.2 | 38.9 | 5.9 |
| Female | 28.2 | 8.7 | 26.3 | 9.1 | 25.4 | 8.7 | 28.1 | 9.0 | 33.0 | 8.3 | 28.1 | 8.5 |
| **Systolic blood pressure, mmHg** | | |  |  |  |  |  |  |  |  |  |  |
| Male | 128.9 | 134.4 | 129.4 | 134.5 | 127.4 | 134.9 | 130.2 | 134.3 | 129.3 | 134.4 | 128.2 | 134.1 |
| Female | 132.2 | 130.5 | 132.8 | 130.1 | 132.4 | 130.0 | 133.0 | 130.6 | 131.9 | 130.4 | 130.7 | 131.5 |
| **Cholesterol, mmol/L** |  |  |  |  |  |  |  |  |  |  |  |  |
| Male | 5.8 | 5.31 | 5.8 | 5.3 | 5.9 | 5.3 | 5.8 | 5.3 | 5.9 | 5.3 | 5.8 | 5.3 |
| Female | 5.9 | 5.4 | 5.9 | 5.4 | 5.9 | 5.4 | 5.9 | 5.4 | 5.9 | 5.4 | 6.0 | 5.5 |
| **Body mass index, kg/m^2^** |  |  |  |  |  |  |  |  |  |  |  |  |
| Male | 26.0 | 26.7 | 25.6 | 26.2 | 25.6 | 26.4 | 26.6 | 26.6 | 26.3 | 26.9 | 26.0 | 27.1 |
| Female | 25.1 | 25.8 | 24.3 | 24.9 | 24.8 | 25.4 | 24.9 | 25.9 | 25.3 | 26.2 | 26.3 | 26.3 |

***** Diabetes data are from the Danish National Patients Registry

|  |
| --- |

# Tables S11. Model fit by age, sex and deprivation quintiles

**Table S11.1 Actual Total CHD deaths prevented or postponed (DPPs) by age, sex and deprivation quintiles (*expected deaths had 1991 rates persisted – observed deaths in 2007)***

|  | **Denmark** | **SECQ1** | **SECQ2** | **SECQ3** | **SECQ4** | **SECQ5** |
| --- | --- | --- | --- | --- | --- | --- |
| **Male** | 6975 | 987 | 1218 | 1368 | 1568 | 1836 |
| **Female** | 4135 | 576 | 638 | 670 | 745 | 1509 |
| **Male 25-34** | 4 | 2 | -2 | 3 | 1 | 0 |
| **Male 35-44** | 38 | 4 | 13 | 14 | 12 | 5 |
| **Male 45-54** | 324 | 46 | 66 | 69 | 59 | 85 |
| **Male 55-64** | 1386 | 183 | 236 | 286 | 371 | 311 |
| **Male 65-74** | 2443 | 322 | 429 | 484 | 558 | 650 |
| **Male 75-84** | 2780 | 429 | 477 | 511 | 568 | 795 |
|  |  |  |  |  |  |  |
|  |  |  |  |  |  |  |
| **Female 25-34** | 1 | 0 | 0 | 2 | 0 | 0 |
| **Female 35-44** | 9 | 4 | 0 | 0 | 0 | 4 |
| **Female 45-54** | 95 | 9 | 19 | 23 | 26 | 18 |
| **Female 55-64** | 511 | 52 | 81 | 122 | 123 | 133 |
| **Female 65-74** | 1108 | 140 | 181 | 198 | 199 | 390 |
| **Female 75-84** | 2410 | 369 | 356 | 324 | 397 | 964 |
|  |  |  |  |  |  |  |
|  |  |  |  |  |  |  |
| **Total** | **11111** | **1561** | **1857** | **2036** | **2313** | **3345** |

**Table S11.2 DPPs estimated by model by age, sex and deprivation quintiles**

|  | **Denmark** | **SECQ1** | **SECQ2** | **SECQ3** | **SECQ4** | **SECQ5** |
| --- | --- | --- | --- | --- | --- | --- |
| **Male** | 4729 | 667 | 817 | 1149 | 978 | 1120 |
| **Female** | 2363 | 287 | 396 | 426 | 608 | 649 |
| **Male 25-34** | 15 | 3 | 1 | 5 | 4 | 2 |
| **Male 35-44** | 68 | 9 | 17 | 16 | 18 | 8 |
| **Male 45-54** | 323 | 41 | 52 | 86 | 64 | 80 |
| **Male 55-64** | 1380 | 196 | 228 | 220 | 377 | 360 |
| **Male 65-74** | 1881 | 288 | 306 | 431 | 304 | 550 |
| **Male 75-84** | 1062 | 130 | 213 | 391 | 211 | 120 |
|  |  |  |  |  |  |  |
| **Female 25-34** | 7 | 1 | 1 | 3 | 1 | 1 |
| **Female 35-44** | 21 | 5 | 3 | 2 | 4 | 7 |
| **Female 45-54** | 116 | 17 | 18 | 17 | 30 | 34 |
| **Female 55-64** | 513 | 53 | 54 | 103 | 154 | 148 |
| **Female 65-74** | 753 | 87 | 146 | 139 | 148 | 232 |
| **Female 75-84** | 954 | 124 | 173 | 161 | 270 | 228 |
|  |  |  |  |  |  |  |
| **Total** | **7092** | **954** | **1213** | **1575** | **1586** | **1769** |

**Table S11.3 Model fit^†^ by age, sex and deprivation quintiles**

|  | **Denmark** | **SECQ1** | **SECQ2** | **SECQ3** | **SECQ4** | **SECQ5** |
| --- | --- | --- | --- | --- | --- | --- |
| **Male** | 68% | 68% | 67% | 84% | 62% | 61% |
| **Female** | 57% | 50% | 62% | 64% | 82% | 43% |
|  |  |  |  |  |  |  |
| **Male 25-34** | 383% | 180% | -39% | 162% | 285% | -396% |
| **Male 35-44** | 177% | 221% | 127% | 117% | 148% | -160% |
| **Male 45-54** | 99% | 89% | 80% | 124% | 108% | 94% |
| **Male 55-64** | 100% | 107% | 97% | 77% | 102% | 116% |
| **Male 65-74** | 77% | 89% | 71% | 89% | 55% | 85% |
| **Male 75-84** | 38% | 30% | 45% | 76% | 37% | 15% |
|  |  |  |  |  |  |  |
| **Female 25-34** | 490% | -578% | -1106% | 177% | -1120% | -1201% |
| **Female 35-44** | 235% | 127% | 2358% | 1655% | 2439% | 154% |
| **Female 45-54** | 121% | 189% | 94% | 74% | 116% | 185% |
| **Female 55-64** | 100% | 102% | 67% | 85% | 126% | 111% |
| **Female 65-74** | 68% | 62% | 81% | 70% | 75% | 59% |
| **Female 75-84** | 40% | 34% | 48% | 50% | 68% | 24% |
|  |  |  |  |  |  |  |
| **Total** | **64%** | **61%** | **65%** | **77%** | **69%** | **53%** |

**^†^** Model fit = absolute % of the total DPPs explained by the model:

***% Model fit = ABSOLUTE (1- ((total DPPs – model DPPs)/total DPPs)) × 100***

**Table S11.4: Overall model fit by deprivation quintile: comparing modelled deaths prevented or postponed (DPPs) against observed fall in CHD deaths, and 95% uncertainty intervals (UI).**

|  | **Target DPPs** | **Explained DPPs** | ***Lower UI***  ***(95%)*** | ***Upper UI***  ***(95%)*** | **Explained (%)** | ***Lower UI (%)*** | ***Upper UI (%)*** |
| --- | --- | --- | --- | --- | --- | --- | --- |
| **SECQ1** | 1561 | 954 | *874* | *1070* | 61 | *56* | *69* |
| **SECQ2** | 1857 | *1213* | *1067* | *1313* | 65 | *57* | *71* |
| **SECQ3** | 2036 | 1574 | *1508* | *1767* | 77 | *74* | *87* |
| **SECQ4** | 2313 | 1586 | *1479* | *1767* | 69 | *64* | *76* |
| **SECQ5** | 3345 | 1769 | *1729* | *2138* | 53 | *52* | *64* |
| **Denmark** | **11111** | **7092** | ***6834*** | ***7869*** | **64** | ***62*** | ***71*** |

# Table S12. Uncertainty analysis: parameter distributions, functions and sources

Table S12 records the type of distribution and associated functions for each of the input variables in the IMPACT_SEC_ model. We implemented uncertainty analysis in Excel using Ersatz (version 1.0 available at <http://www.epigear.com>), an add-in that allows probabilistic bootstrapping in Excel.(57) Ersatz allows repeated random draws from specified distributions for input variables and then calculates the 95% uncertainty intervals from the realised values of the output variable (deaths prevented or postponed). For the IMPACT_SEC_ model, we calculated the uncertainty intervals based on 1000 draws taking the 95% uncertainty intervals as the 2.5^th^ and 97.5^th^ percentiles. Input variables taken from external sources (e.g. case fatality rates, beta coefficients and relative risk reductions) were randomly drawn from specified distributions but assumed constant across deprivation quintiles. Worked examples using Ersatz to estimate uncertainty intervals for net treatment DPPs and DPPs attributable to risk factor change are shown below Table S12.1.

Table S12.1. Input parameters, type of distribution and sources for data variables used in the sensitivity analyzes

| **Input parameters** | ***Type of distribution and functions (Mean, Standard error)*** | Source |
| --- | --- | --- |
| **Population** | | |
| Population counts and CHD deaths stratified by age, gender, and Socio-economic quintiles based on financial income. | - Population counts (no error) - Deaths expected in 2007 had CHD mortality rates in 1991 persisted (***Poisson distribution***) | Central Office of Civil Registration, Cause of Death(21) Registry and Statistic Denmark |
| **Risk factors** | | |
| Prevalence/mean estimates (pooled data; national estimates for 1991 and 2007) | - Prevalence estimates (smoking, physical activity, diabetes): (***Beta distribution***: cases, sample-size minus cases) - Continuous variables (Body Mass Index, SBP, Total cholesterol,): (***Normal distribution***: mean, SE of mean) | Copenhagen City Heart Study 3 and 4(7) |
| RR: **smoking** | ***Ersatz RR function*** (RR, SE ln(RR)):  RRs and 95% CIs shown in Table S9. | Ezzati et al (2005)(52) |
| RR: **physical inactivity** | ***Ersatz RR function*** (RR, SE ln(RR)):  RRs and 95% CIs shown in Table S9. | Bull et al (2004)(53) |
| RR: **diabetes** | ***Ersatz RR function*** (RR, SE ln(RR)):  RRs and 95% CIs shown in Table S9. | Roglic and Unwin(2010), and Huxley et al (2006)(12,13) |
| Beta coefficient: **Body Mass Index** | ***Normal distribution*** (mean, SE of mean):  M & F < 45 (0.036,0.005); M & F 45-54 (0.030, 0.004)  M & F 55-64 (0.023,0.003); M & F 65-74 (0.015, 0.002)  M & F 75-84 (0.012,0.002); M & F 85+ (0.010, 0.001) | Bogers et al (2006), James et al (2004)(50,51). Parameters on the log scale. |
| Beta coefficient: **SBP** | ***Normal distribution*** (mean, SE of mean):  M < 45 (-0.036,0.004); M 45-54 (-0.035,0.004)  M 55-64 (-0.032,0.003); M 65-74 (-0.027,0.003)  M 75-84 (-0.021,0.002); M 85+ (-0.016,0.002)  F < 55 (-0.046, 0.005); F 55-64 (-0.035,0.004)  F 65-74 (-0.032,0.003); F 75-84 (-0.026,0.003)  F 85+ (-0.019,0.002) | Prospective studies collaborative meta-analysis (2002)(49). Parameters on the log scale. |
| Beta coefficient: **Total cholesterol** | ***Normal distribution*** (mean, SE of mean):  M < 45 (-0.799,0.081); M 45-54 (-0.755,0.077)  M 55-64 (-0.446,0.046); M 65-74 (-0.236,0.024)  M 75-84 (-0.117,0.012); M 85+ (-0.083,0.009)  F < 45 (-0.844,0.086); F 45-54 (-0.734,0.075)  F 55-64 (-0.431,0.044); F 65-74 (-0.261,0.027)  F 75-84 (-0.174,0.018); F 85+ (-0.051,0.005) | Prospective studies collaborative meta-analysis (2007)(9). Parameters on the log-scale. |
| **ST elevation myocardial infarction (STEMI)** | | |
| **Eligible patients**:  Hosptial discharge diagnosis of myocardial infarction. (No detailed data on STEMI and nSTEMI in 1991 so the STEMI/nSTEMI ratio for all myocardial infarction was assumed to be 40/60) | ***Poisson distribution*** (admissions) | Danish National Patient Registry (DNPR) |
| **Case fatality rate** | Sample size (*n*) = STEMI admissions:  ***Beta distribution*** (cases = *n* × CFR estimate^5^, non-cases = *n* – cases) | Wijeysundera et al (2010)(5) |
| **Treatment uptake** | - *Medications:* ***Beta distribution*** (cases = STEMI discharge diagnosis from DNPR × medication uptake, non-cases = STEMI admissions – cases) - *PCI and CABG*: ***Beta distribution*** (cases = MI discharge diagnosis from DNPR × PCI/CABG uptake, non-cases = MI admissions – cases) | Survey from region of Northern Jutland (2007) and Prescription Registry Fuen County (1991), DNPR (PCI/ CABG) . |
| **Relative risk reduction:** | ***Ersatz RR function*** (RRR, SE ln(RRR)): |  |
| Thrombolysis  Aspirin  Beta-blockers  Primary PCI  Primary CABG surgery  ACE Inhibitors | M & F (0.31,0.298)  M & F (0.23,0.177)  M & F (0.04,0.691): assumed lower limit of 1%  M & F (0.30,0.587)  M & F (0.39,0.293)  M & F (0.07,0.435) | Estess (2002)  ISIS-2 (1998)  Freemantle (1999)  Keeley (2003)  Yusuf (1994)  ACE-I MI Collaborative Group (1998)(24-29) |
| Clopidogrel | M & F (0.03,0.457) | Chen (2003) and Sabatine (2005)(30,31) |
| **Non-ST segment elevation acute coronary syndrome (NSTEACS)** | | |
| **Eligible patients**:  Hospital discharge diagnosis of myocardial infarction (ratio of STEMI/nSTEMI assumed to be 40/60)(15) or primary diagnosis of unstable angina | ***Poisson distribution*** (nSTEMI + unstable angina admissions) | DNPR |
| **Case fatality rate** | Sample size (*n*) = nSTEMI + unstable angina admissions:  ***Beta distribution*** (cases = *n* × CFR estimate^5^, non-cases = *n* – cases) | Wijeysundera et al (2010)(5) |
| **Treatment uptake** | - *Medications:* ***Beta distribution*** (cases = NSTEACS discharge diagnosis from DNPR × medication uptake, non-cases = NSTEACS admissions – cases) - *PCI and CABG*: ***Beta distribution*** (cases = NSTEACS from DNPR × PCI/CABG uptake, non-cases = unstable angina admissions – cases) | Survey from region of Northern Jutland 2007, Prescription Registry Fuen county (1991) DNPR (PCI/ CABG). |
| **Relative risk reduction:** | ***Ersatz RR function*** (RRR, SE ln(RRR)): |  |
| Aspirin & heparin  Primary CABG surgery  Early PCI  Beta blockers  Clopidogrel  ACE Inhibitors | M & F (0.33,0.470)  M & F (0.39,0.293)  M & F (0.32,0.592)  M & F (0.04,0.691): assumed lower limit of 1%  M & F (0.07,0.435)  M & F (0.07,0.435) | Oler (1996)  Yusuf (1994)  RITA 3 (Fox 2005)  Freemantle (1999)  Yusuf (2001)  ACE-I MI Collaborative Group (1998) (26,28,29,33,35,36) |
| Aspirin alone | M & F (0.15,0.139) | Antithrombotic Trialists’ Collaboration ATC (2002)(32) |
| Platelet glycoprotein IIB/IIIA inhibitors | M & F (0.09,0.530) | Boersma (2002)(34) |
| **Secondary prevention post myocardial infarction (MI)** | | |
| **Eligible patients**:  Having had a myocardial infarction in 10 previous years (excluding cases with HF and incident CHD in index year) | ***Poisson distribution*** (Population in 2007 × (post-MI prevalence obtained from DNPR) | DNPR |
| **Case fatality rate** | Sample size (*n*) = ever having had MI in LPR in 2007:  ***Beta distribution*** (cases = *n* × CFR estimate^5^, non-cases = *n* – cases) | Wijeysundera et al (2010)(5) |
| **Treatment uptake** | ***Beta distribution*** (cases = *n* × medication uptake, non-cases = *n* – cases) | Prescription Registry Fuen (1991) and Danish Medical Agency (2007) |
| **Compliance** | Sample size (*n1*) = having had MI previous 10 years in DNPR (excluding cases with HF and incident CHD in 2007):  ***Beta distribution*** (cases = *n1* × assumed compliance, non-cases = *n1* – cases) |  |
| **Relative risk reduction:** | ***Ersatz RR function*** (RRR, SE ln(RRR)): |  |
| Aspirin  Beta blockers  ACE Inhibitors  Statins  Warfarin | M & F (0.15,0.139)  M & F (0.23,0.185)  M & F (0.20,0.177)  M & F (0.24,0.245)  M & F (0.22,0.305) | ATC (2002)  Freemantle (1999)  Flather (2000)  Hulten (2006)  Anand and Yusuf (1999)(28,32,38,39) |
| **Secondary prevention post revascularisation** | | |
| **Eligible patients**:  Having had a revascularisation procedure previous 10 years, and assumed be alive prior to index years(excluding cases with HF, MI and incident CHD in 2007) | - ***Poisson distribution*** (CABG/PCI procedures from 1991 to 2007 | DNPR |
| **Case fatality rate** | Sample size (*n*) = ever having had post-revascularisation in GPRD in 2007:  ***Beta distribution*** (cases = *n* × CFR estimate^5^, non-cases = *n* – cases) | Wijeysundera et al (2010)(5) |
| **Treatment uptake** | ***Beta distribution*** (cases = *n* × medication uptake, non-cases = *n* – cases) | Prescription Registry Fuen (1991) and Danish Medical Agency (2007) |
| **Compliance** | Sample size (*n1*) = having had revascularisation in DNPR or Danish Heart Registry from previous 11 years:  ***Beta*** ***distribution*** (cases = *n1* × assumed compliance, non-cases = *n1* – cases) |  |
| **Relative risk reduction:** | ***Ersatz RR function*** (RRR, SE ln(RRR)): |  |
| Aspirin  Beta blockers  ACE Inhibitors  Statins  Rehabilitation  Warfarin | M & F (0.15,0.139)  M & F (0.23,0.185)  M & F (0.20,0.177)  M & F (0.24,0.245)  M & F (0.26,0.347)  M & F (0.22,0.305) | ATC (2002)  Freemantle (1999)  Flather (2000)  Hulten (2006)  Taylor (2004)  Anand and Yusuf (1999)(16,28,32,37,39) |
| **Chronic stable coronary artery disease** | | |
| **Eligible patients**:  Having had chronic stable artery disease previous 10 years (prior to index years (excluding cases with HF, MI, previous revascularisation and incident CHD in 2007) | ***Poisson*** ***distribution*** (Population in 2007 × (chronic stable coronary artery disease) | DNPR |
| **Case fatality rate** | Sample size (*n*) = having had angina but no HF, MI or previous revascularisation previous 10 years:  ***Beta distribution*** (cases = *n* × CFR estimate^5^, non-cases = *n* – cases) | Wijeysundera et al (2010)(5) |
| **Treatment uptake** | ***Beta distribution*** (cases = *n* × medication uptake, non-cases = *n* – cases) | Prescription Registry Fuen (1991) and Danish Medical Agency (2007) |
| **Compliance** | Sample size (*n1*) = having had angina but no HF, MI or previous revascularisation previous 10 years  ***Beta distribution*** (cases = *n1* × assumed compliance, non-cases = *n1* – cases) |  |
| **Relative risk reduction:** Statins  Aspirin  ACE Inhibitors | ***Ersatz RR function*** (RRR, SE ln(RRR)):  M & F (0.23,0.244)  M & F (0.15,0.139)  M & F (0.17,0.177) | Wilt (2004)  ATC (2002)  Al-Mallah (2006)  (32,41,42) |
| **CABG for chronic stable coronary artery disease** | | |
| **Eligible patients**:  Having had a revascularisation procedure previous 10 years, and assumed be alive prior to index years(excluding cases with HF, MI and incident CHD in 2007) | - ***Poisson distribution*** (patients having had a CABG) | DNPR |
| **Case fatality rate** | As described in the post-revascularisation group |  |
| **Treatment uptake and compliance** | Fixed at 100% |  |
| **Relative risk reduction:** CABG (0-5 years) | ***Ersatz RR function*** (RRR, SE ln(RRR)):  M & F (0.39,0.293) | Yusuf (1994)(26) |
| **Heart failure in patients requiring hospitalisation** | | |
| **Eligible patients**:  Hospital discharge with a primary diagnosis of heart failure | ***Poisson distribution*** (HF discharge minus 50% assumed not to be due to CHD) | DNPR |
| **Case fatality rate** | Sample size (*n*) = DNPR discharge for HF divided by 2 as only half assumed to be CHD related  ***Beta distribution*** (cases = *n* × CFR estimate^5^, non-cases = *n* – cases) | Wijeysundera et al (2010)(5) |
| **Treatment uptake** | Medications: Sample size (*n1*) = HF discharge diagnosis from survey from region of Northern Jutland and Prescription Registry Fuen County:   - ***Beta distribution*** (cases = *n1* × medication uptake, non-cases = *n1* – cases) | Prescription Registry Fuen (1991) and Danish Medical Agency (2007) |
| **Compliance** | Sample size (*n2*) = HF discharge diagnosis from survey from region of Northern Jutland and Prescription Registry Fuen County with record of medication use:  ***Beta distribution*** (cases = *n2* × assumed compliance, non-cases = *n2*  – cases) | Prescription Registry Fuen (1991) and Danish Medical Agency (2007) |
| **Relative risk reduction:** Aspirin  ACE Inhibitors  Beta blockers  Spironolactone | ***Ersatz RR function*** (RRR, SE ln(RRR)):  M & F (0.15,0.139)  M & F (0.20,0.177)  M & F (0.35,0.128)  M & F (0.30,0.128) | ATC (2002)  Flather (2000)  Shibata (2001)  Pitt (1999)  (32,37,43,44) |
| **Heart failure in the community** | | |
| **Eligible patients**:  Having had heart failure 10 previous years but no incident HF in index years | ***Poisson distribution*** (Population in 2007 × HF prevalence obtained from DNPRdivided by 2 as only half assumed to be CHD related) and no incident HF hospital admissions) | DNPR |
| **Case fatality rate** | Sample size (*n*) = having had HF in DNPR previous 10 years:  ***Beta distribution*** (cases = *n* × CFR estimate^5^, non-cases = *n* – cases) | Wijeysundera et al (2010)(5) |
| **Treatment uptake** | ***Beta distribution*** (cases = *n* × medication uptake, non-cases = *n* – cases) | Prescription Registry Fuen (1991) and Danish Medical Agency (2007) |
| **Compliance** | Sample size (*n1*) = having had HF in DNPR previous 10 years with registration of medication use:  ***Beta distribution*** (cases = *n1* × assumed compliance, non-cases = *n1* – cases)^6^ |  |
| **Relative risk reduction:** Aspirin  ACE Inhibitors  Beta blockers  Spironolactone | ***Ersatz RR function*** (RRR, SE ln(RRR)):  M & F (0.15,0.139)  M & F (0.20,0.177)  M & F (0.35,0.128)  M & F (0.31,0.216) | ATC (2002)  Flather (2000)  Shibata (2001)  Pitt (1999)  (32,37,43,44) |
| **Primary prevention therapies: Statins** | | |
| **Eligible patients**:  Population | Population counts (no error) | The Central Office of Civil Registration(21) |
| **Treatment uptake** | % No CHD and registrated to have had collected prescribed lipid lowering drugs: (***Beta distribution***: cases, sample-size minus cases) | CCHS 3 and 4, Prescription Registry Fuen (1991) and Danish Medical Agency (2007) |
| **Case fatality rate** | Sample size (*n*) = no known CHD and registrated to have had collected prescribed lipid lowering medicine in the index years:  ***Beta distribution*** (cases = *n* × CFR estimate^5^, non-cases = *n* – cases) | Wijeysundera et al (2010)(5) |
| **Compliance** | ***Beta distribution*** (cases = *n* × assumed compliance, non-cases = *n* – cases) | Prescription Registry Fuen (1991) and Danish Medical Agency (2007) |
| **Relative risk reduction:** Statins | ***Ersatz RR function*** (RRR, SE ln(RRR)):  M & F (0.35,0.396) | Pignone (2000)(48) |
| **Primary prevention therapies: Treatments for high blood pressure** | | |
| **Eligible patients**:  Population | Population counts (no error) | The Central Office of Civil Registration(21) |
| **Treatment uptake** | % no known CHD and registrated to have had collected prescribed antihypertensive medicine: (***Beta distribution***: cases, sample-size minus cases) | CCHS 3 and 4(7), Prescription Registry Fuen (1991) and Danish Medical Agency (2007) |
| **Case fatality rate** | Sample size (*n*) = no known CHD and registrated to have had collected prescribed antihypertensive medicine in the index years:  ***Beta distribution*** (cases = *n* × CFR estimate^5^, non-cases = *n* – cases) | Wijeysundera et al (2010)(5) |
| **Compliance** | ***Beta distribution*** (cases = *n* × assumed compliance, non-cases = *n* – cases) | Prescription Registry Fuen (1991) and Danish Medical Agency (2007) |
| **Relative risk reduction:** Treatments for high blood pressure | ***Ersatz RR function*** (RRR, SE ln(RRR)):  M & F (0.13,0.294) | Law (2003)(47) |

**WORKED EXAMPLES USING ERSATZ FOR UNCERTAINTY ANALYSIS**

Below we illustrate our use of Ersatz for uncertainty analysis using males aged 75-84 in the most deprived quintile.

**1 Uncertainty analysis for treatments**

The net effects of aspirin in the secondary prevention post MI group (DG3) was calculated as follows:

***DPPs (2007) = patient numbers × treatment uptake_2007_ × compliance × relative mortality reduction × 1-year case fatality***

= (1,546) × 0.76 × 0.70 × 0.15 × 0.067 ≈ 8.3

***DPPs (1991) = patient numbers × treatment uptake_1991_ × compliance × relative mortality reduction × 1-year case fatality***

= (1,546) × 0.06 × 0.70 × 0.15 × 0.067 ≈ 0.7

Net DPPs were therefore calculated as = **DPPs_2007_ – DPPs_1991_** ≈ 8.3 – 0.7 ≈ 7.6

Table S12 above shows the probability distributions and associated Ersatz functions used for each input variable in the DPP calculations. For secondary prevention post MI we used Poisson (patient numbers); beta (treatment uptake, compliance, and case fatality rate) and the Ersatz RR function (relative risk reduction in the 1-year case fatality rate owing to treatment). More specifically, for males aged 75-84 in secq5, the input values for the uncertainty analysis were as follows:

- Patient numbers ~ Poisson (population in 2007 × post-MI prevalence) ~ Poisson (1,546)
- Treatment uptake in 2007 and 1991 ~ Beta (cases, non-cases)

If we let *n* denote ever having had MI in 1991-2007 then cases = (*n* × uptake of aspirin) and non-cases = (*n* – cases). Cases = (1,546 × 0.76) = 1175; non-cases = (1546-1175) = 371. Treatment uptake in 2007 was therefore ~ Beta (1175,371). Treatment uptake in 1991 was ~ Beta (93,1453).

- Compliance ~ Beta (cases, non-cases)

If we let *n1* denote ever having had MI in 2007 *and* with record of medication use in 2007 then cases = (*n1* × assumed compliance (0.70)) and non-cases = (*n1* – cases). Cases = (1175 × 0.70) = 823; non-cases = (1175-823) = 352. Compliance therefore ~ Beta (823,352)

- Relative risk reduction (RRR) ~ Ersatz RR function (RRR, SE ln(RRR))

where SE and ln denote standard error and natural logarithm, respectively. RRR was 1-odds ratio for aspirin use in the community taken from The Danish Medical Agency = 1-0.76 = 0.24; with 95% Confidence Interval (0.11,0.19)(32). Using the 95% CIs, the SE of ln(RR) was calculated as:

ln(0.19)-ln(0.11)/(1.96×2) = 0.139

Relative risk reduction for aspirin was therefore ~ Ersatz RR function (0.15,0.139)

- Case fatality rate ~ Beta (cases, non-cases)

Parameter uncertainty around case fatality rates was calculated at the national level and assumed constant across all sec quintiles. If we let *n* denote ever having had MI 1991-2007 in Denmark then cases = (*n* × assumed CFR (0.067)) and non-cases = (*n* – cases). Using the DNPR-data for males aged 75-84 and assumed CFRs(5), cases = (7492 × 0.067) = 502; non-cases = (7492-502) = 6990. Case fatality for the post-MI group was therefore ~ Beta (502, 6990).

Putting all this together, 10 runs in Ersatz gave the following estimates of each input variable randomly drawn from the relevant probability distributions from which we calculate the net DPPs for aspirin in males aged 75-84 in secq5:

**Table S12.2 10 runs in Ersatz to calculate net DPPs for aspirin use in males aged 75-84 in SECQ5 (post-MI)**

| **Run** | **Numbers** | **Uptake_2007_** | **Compliance** | **Relative risk reduction** | **Case fatality rate** | **Uptake_1991_** | **Net DPPs†** |
| --- | --- | --- | --- | --- | --- | --- | --- |
|  | **Probability distributions** | | | | | | |
|  | **Poisson** | **Beta** | **Beta** | **RR** | **Beta** | **Beta** |  |
|  | **Col A** | **Col B** | **Col C** | **Col D** | **Col E** | **Col F** |  |
| 1 | 1534 | 0.762 | 0.708 | 0.124 | 0.068 | 0.063 | 6.40 |
| 2 | 1497 | 0.773 | 0.705 | 0.168 | 0.067 | 0.066 | 8.40 |
| 3 | 1593 | 0.778 | 0.708 | 0.11 | 0.075 | 0.063 | 6.65 |
| 4 | 1479 | 0.748 | 0.726 | 0.172 | 0.069 | 0.061 | 8.75 |
| 5 | 1503 | 0.754 | 0.708 | 0.155 | 0.066 | 0.063 | 7.52 |
| 6 | 1478 | 0.753 | 0.692 | 0.13 | 0.07 | 0.054 | 6.51 |
| 7 | 1469 | 0.749 | 0.694 | 0.166 | 0.068 | 0.062 | 7.91 |
| 8 | 1596 | 0.744 | 0.696 | 0.168 | 0.064 | 0.072 | 8.03 |
| 9 | 1569 | 0.779 | 0.707 | 0.139 | 0.072 | 0.055 | 8.04 |
| 10 | 1567 | 0.758 | 0.698 | 0.181 | 0.065 | 0.065 | 8.92 |
| **Point estimate** | **1546** | **0.76** | **0.7** | **0.15** | **0.067** | **0.06** | **7.71** |

^†^ Net DPPs = (A×B×C×D×E) - (A×F×C×D×E)

*Uncertainty intervals for treatment contribution DPPs*

In each Ersatz run, net DPPs were calculated for all age-sex-secq groups and were then summed for each medication within each of the 9 mutually exclusive CHD patient groups. Each of the nine treatment DPP totals was then multiplied by a correction for polypharmacy (which varied across patient groups but took the same value in each of the 1000 Ersatz runs). An estimate of the total treatment contribution to model DPPs was obtained by summing the nine patient group totals.

95% uncertainty intervals from the set of 1000 runs (2.5^th^ and 97.5^th^ percentiles) were extracted for the nine treatment totals plus the overall estimate of treatment contribution DPPs. These are shown in Table S2.

**2 Uncertainty analysis for change in binary risk factors**

The DPPs attributable to change in smoking prevalence over 1991-2007 was calculated as follows:

***DPPs = expected CHD deaths in 2007 (had 1991 mortality rates remained constant) × (PARF_1991_ – PARF_2007_)***

where PARF = [P × (RR-1)] / [1 + P × (RR-1)]; P is the prevalence of the risk factor and RR is

the relative risk for CHD mortality associated with risk factor presence.

For the three binary risk factors (smoking, diabetes, and physical activity) we used the following probability distributions: Poisson (expected deaths); beta (risk factor prevalence) and the Ersatz RR function (relative risk reduction owing to elimination of exposure). More specifically, for males aged 75-84 in secq5, the input values for the uncertainty analysis were as follows:

- Expected CHD deaths in 2007 ~ Poisson (population in 2007 × CHD mortality rates in 1991) ~ Poisson (1080)
- Estimates of smoking prevalence ~ Beta (cases, non-cases)

If we let *n* denote the number of CCHS male respondents 2007 then cases = (*n* × estimate of smoking prevalence) and non-cases = (*n* – cases). Cases = (2500 × 0..42) = 1050; non-cases = (2500-1050) = 1450. Smoking prevalence over 1991-2007 therefore ~ Beta (1050,1450). The same method was used for a pooled estimate of smoking prevalence at the national level.

National estimates of smoking prevalence were calculated in the start and final years of the model (1991 and 2007). Smoking prevalence in Denmark for males aged 75-84 in 1991 ~ Beta (22,225); smoking prevalence in Denmark for males aged 75-84 in 2007 ~ Beta (26,216)

- Increased relative risk attributable to smoking ~ Ersatz RR function (RR, SE ln(RR))

where RR was taken from the CPS-II(52) study. For males aged 75-84, RR for smoking = 1.31 with 95% CI (1.11,1.56). Using the 95% CI the SE of ln(RR) was calculated as follows:

ln(1.56)-ln(1.11)/(1.96×2) = 0.088

The relative risk for smoking in males aged 75-84 was therefore ~ Ersatz RR function (1.31, 0.088)

Putting all this together, 10 runs in Ersatz gave the following estimates of each input variable randomly drawn from the relevant probability distributions from which we calculate DPPs from the change in smoking prevalence over 1991-2007 in males aged 75-84 in secq5:

**Table S12.3 10 runs in Ersatz to calculate DPPs for change in smoking prevalence over 1991-2007 in males aged 75-84 in secq5**

| **Run** | **Expected deaths** | **Relative risk** | **% smoke in 1991_IMD_** | **% smoke in 2007_IMD_** | **DPPs†** |
| --- | --- | --- | --- | --- | --- |
| **Probability distributions** | | | | | |
|  | **Poisson** | **RR** | **Beta** | **Beta** |  |
|  | **Col A** | **Col B** | **Col C** | **Col D** |  |
| 1 | 1104 | 1.31 | 0.562 | 0.162 | -111 |
| 2 | 1097 | 1.37 | 0.612 | 0.158 | -142 |
| 3 | 1118 | 1.36 | 0.545 | 0.067 | -157 |
| 4 | 1140 | 1.30 | 0.587 | 0.116 | -132 |
| 5 | 1105 | 1.30 | 0.560 | 0.206 | -95 |
| 6 | 1053 | 1.21 | 0.581 | 0.198 | -72 |
| 7 | 1065 | 1.40 | 0.622 | 0.171 | -144 |
| 8 | 1003 | 1.32 | 0.531 | 0.219 | -80 |
| 9 | 1102 | 1.35 | 0.544 | 0.245 | -89 |
| 10 | 1124 | 1.41 | 0.569 | 0.123 | -159 |
| **Point estimate** | **1080** | **1.31** | **0.588** | **0.181** | **-109** |

† Intermediate steps in calculating DPPs as follows:

PARF in 1991 (secq) = (C × (B-1))/(C × (B-1)+1);

PARF in 2007 (secq) = (D × (B-1))/(D × (B-1)+1)

DPPs = A × ((PARF in 1991 (secq)) - ((PARF in 2007 (secq))

*Uncertainty intervals for binary risk factor DPPs*

Within each run, DPPs were calculated for all age-gender-sec groups and were then summed within each of the three binary risk factors. 95% uncertainty intervals from the set of 10000 runs (2.5^th^ and 97.5^th^ percentiles) were extracted for each risk factor total. The 95% uncertainty intervals were then scaled down using an overall correction for cumulative risk reduction (See Section 1.3). These adjusted 95% uncertainty intervals are shown in Table S3.

**3 Uncertainty analysis for change in continuous risk factors**

The DPPs attributable to change in mean levels of systolic blood pressure (SBP) over 1991-2007 was calculated as follows:

***DPPs = expected CHD deaths in 2007 (had 1991 mortality rates remained constant) × absolute risk factor reduction between 1991 and 2007 × regression coefficient exponentiated***

For continuous risk factors (SBP, total cholesterol, and BMI) we used the Poisson (expected deaths) and Normal probability distributions (mean risk factor levels and beta coefficients). The input values for the uncertainty analysis were as follows:

- Expected deaths ~ Poisson (population in 2007 × CHD mortality rates in 1991) ~ Poisson (4236)
- Estimates of SBP levels ~ Normal (mean, SE mean)

For males aged 75-84 in secq5 mean SBP levels using CCHS 3 and 4 data for 1991 and 2007 ~ Normal (mean = 143.1 mmHg, SE = 1.51). The SE was estimated in Stata version 11.1 to account for the complex survey design. Likewise, for males aged 75-84 in Denmark mean SBP levels over 1991-2007 ~ Normal (mean = 141.0, SE = 0.49).

National estimates for 1991 and 2007 were used to estimate absolute change in SBP over the sixteen year period. In 1991, mean SBP for males aged 75-84 in Denmark was ~ Normal (mean = 146.4, SE = 1.39); for 2007 mean SBP ~ Normal (mean = 159.7, SE = 1.48).

- Beta coefficients ~ Normal (mean, SE)

where the beta coefficient for SBP (on the logarithmic scale) taken from the PSC study for males aged 75-84 was -0.0212 with 95% CI (-0.0170,-0.0255)(49). Using the 95% CIs the SE of the beta coefficient was calculated as follows:

(-0.0170) - (-0.0255)/(1.96×2) = 0.0022

The beta coefficient for SBP for males aged 75-84 in secq5 was therefore ~ Normal (mean = -0.0212, SE = 0.0022).

Putting all this together, 10 runs in Ersatz gave the following estimates of each input variable drawn from the relevant probability distributions from which we calculated DPPs from the change in mean SBP over 1991-2007 in males aged 75-84 in secq5:

**Table S12.4 10 runs in Ersatz to calculate DPPs for change in mean SBP levels over 1991-2007 in males aged 75-84 in secq5**

| **Run** | **Expected deaths** | **Beta** | **SBP in 1991_Den_** | **SBP in 2007_Den_** | **DPPs†** |
| --- | --- | --- | --- | --- | --- |
| **Probability distributions** | | | | | |
|  | **Poisson** | **Normal** | **Beta** | **Beta** |  |
|  | **Col A** | **Col B** | **Col C** | **Col D** |  |
| 1 | 1104 | -0.0194 | 147.0 | 160.5 | -254 |
| 2 | 1097 | -0.0228 | 146.3 | 160.9 | -311 |
| 3 | 1118 | -0.0243 | 144.8 | 157.9 | -305 |
| 4 | 1140 | -0.0205 | 145.7 | 160.4 | -297 |
| 5 | 1105 | -0.0223 | 146.6 | 159.9 | -284 |
| 6 | 1053 | -0.0209 | 145.5 | 161.4 | -298 |
| 7 | 1065 | -0.0254 | 146.3 | 159.2 | -298 |
| 8 | 1003 | -0.0231 | 148.5 | 159.8 | -230 |
| 9 | 1102 | -0.0221 | 143.9 | 161.2 | -350 |
| 10 | 1124 | -0.0207 | 148.3 | 160.4 | -249 |
| **Point estimate** | 1080 | **-0.0212** | **146.4** | **159.7** | -265 |

† Intermediate steps in calculating DPPs as follows:

Absolute change in mean SBP= abs(D-C)

DPPs = (1-exp(B × (absolute change in mean SBP)) × A

*Uncertainty intervals for continuous risk factor DPPs*

Within each run, DPPs were calculated for all age-sex-secq groups and were then summed within each of the four continuous risk factors. For systolic blood pressure and total cholesterol we then subtracted the DPPs calculated in the treatment (primary prevention) component of the model from the DPPs calculated in the risk factor component (See Section 4.3). 95% uncertainty intervals were then calculated from the set of 1000 runs (taken from the 2.5^th^ and 97.5^th^ percentiles) for the four continuous risk factor DPP totals. These were then scaled down using an overall correction for cumulative risk reduction. The 95% uncertainty intervals for risk factor DPPs are shown in Table S3.

*Uncertainty intervals for total risk factor contribution DPPs*

Within each run, the total risk factor contribution to model DPPs was obtained by summation of seven risk factor DPPs totals (within each age-sex-secq group). A 95% uncertainty interval for the overall risk factor contribution was obtained from the set of 10000 runs by taking the 2.5^th^ and 97.5^th^ percentiles. These lower and upper limits were then scaled down using the correction for cumulative risk reduction. The 95% uncertainty intervals for total risk factor contribution DPPs are shown in Table S3.

*Uncertainty Intervals for total model DPPs (changes in treatment uptake and risk factors)*

Within each run, we also calculated an estimate of the total model DPPs by summation of the treatment contribution DPPs and the risk factor contribution DPPs *after* adjustment for cumulative risk reduction. The standard deviation of the estimated total model DPPs over the set of 10000 runs was extracted for Denmark and each deprivation quintile. The 95% uncertainty intervals for total model DPPs were then obtained as follows:

Lower limit = estimate – (1.96 × standard deviation)

Upper limit = estimate + (1.96 × standard deviation)

The 95% uncertainty intervals for total model DPPs are shown in Table S2.

# Annex SA.

**Calculation of national rates and gradients for treatment uptake and risk factors based on regional registries and population surveys**

Calculation of gradients

The rates of treatment uptake and risk factors were based on various subpopulations. To cope with the sometimes sparse *sex*secq*age* strata a smoothing approach was taken.

The most general smoothing in which the gradient along *secq* and along *age* varies according to a parabola <second order polynomial> is of the form

1. **µ_ij_ = α_0_ + α_1_ (i-3)^2^ + α_2_(j-3)^2^ + α_3_(i-3)(j-3)** i= agegroup; j= secq group

When sparsity was particular pronounced the gradient was assumed to be linear and constant over secq groups

1. **µ_ij_ = α_0_ + α_1_ (i-3)** i= agegroup; j= secq group

Treatment uptakes

*Community treatment uptake*

In 1991 there was no national register for medicine. For 1991 community treatment uptakes data we used the data from the Fuen Prescription Register, including information of all prescriptions on an individual level for the inhabitants of the county of Fuen, (approximately 10% of the Danish population). All prescriptions were linked to the personal 10 digit code, CPR number, and was linked to the main database in Statistic Denmark with information of financial income and diagnoses. Thus we could calculate the treatment uptake rates for the specific disease groups:

ASA treatment uptake ratio for MI patients=(subjects from Fuen with MI taking ASA)/ inhabitants of Fuen)

for each age- gender group for the specific diagnoses. Because some of these groups were small we assumed that the uptake was similar in all sec groups and calculated age gradients where we weighted the uptake rates according to the number of persons in the specific age-gender group.

For 2007 we used the Danish Medical Agency register covering the prescription for the total Danish population.

*In-hospitaltreatment uptake*

In Denmark no register exist with data of in-hospital treatment. We obtained data on in-hospital treatment for angina pectoris, acute coronary syndrome and congestive heart failure through a survey. For each of these diagnosis 500 patients were randomly selected among patients admitted to a hospital department in the region of Northern Jutland in 2007. All medical files were reviewed to ensure that the patients fulfilled the criteria for the respective diagnosis, and if so we registered the in-hospital (medical and invasive) treatment uptake ratios. Because of sparse data for some of the age-sec-gender groups we used “smoothening” as described above.

Risk factors

Data of distribution of risk factors were obtained from population surveys CCHS studies 3 (1993) and 4 (2003).(7) Since CCHS studies did not have data from the index years (1991 and 2007) we extrapolated the changes from CCHS studiy years (1993 and 2003) to the index years using the following formulas:

RF_1991_= RFvalue_1993_ – ((RF_2003 -_  RFvalue_1993_)/10)x2

RF_2007_= RFvalue_2003_ + ((RF_2003 -_  RFvalue_1993_)/10)x4

assuming that the change in RF was linear (that is the annual change rate for the previous 2 year and subsequent 4 years was the same as the mean annual change from CCHS 3 to CCHS 4. We also used the same principles for “smoothening” data as mention above for the treatment uptakes.

# Reference List

1. Unal B, Critchley JA, Capewell S. Explaining the decline in coronary heart disease mortality in England and Wales between 1981 and 2000. *Circulation* 2004;**109**:1101-1107.

2. Ford ES, Ajani UA, Croft JB, Critchley JA, Labarthe DR, Kottke TE, Giles WH, Capewell S. Explaining the decrease in U.S. deaths from coronary disease, 1980-2000. *N Engl J Med* 2007;**356**:2388-2398.

3. Capewell S, Beaglehole R, Seddon M, McMurray J. Explanation for the decline in coronary heart disease mortality rates in Auckland, New Zealand, between 1982 and 1993. *Circulation* 2000;**102**:1511-1516.

4. Capewell S, Morrison CE, McMurray JJ. Contribution of modern cardiovascular treatment and risk factor changes to the decline in coronary heart disease mortality in Scotland between 1975 and 1994. *Heart* 1999;**81**:380-386.

5. Wijeysundera HC, Machado M, Farahati F, Wang X, Witteman W, van der Velde G, Tu JV, Lee DS, Goodman SG, Petrella R, O'Flaherty M, Krahn M, Capewell S. Association of temporal trends in risk factors and treatment uptake with coronary heart disease mortality, 1994-2005. *JAMA* 2010;**303**:1841-1847.

6. Yeh RW, Sidney S, Chandra M, Sorel M, Selby JV, Go AS. Population trends in the incidence and outcomes of acute myocardial infarction. *N Engl J Med* 2010;**362**:2155-2165.

7. Aguib Y, Al Suwaidi J. The Copenhagen City Heart Study (Osterbroundersogelsen). *Glob Cardiol Sci Pract* 2015;**2015**:33.

8. The cohorts at the Research Centre for Prevention and Health, formerly &.

9. Prospective Studies Collaboration, Lewington S, Whitlock G, Clarke R, Sherliker P, Emberson J, Halsey J, Qizilbash N, Peto R, Collins R. Blood cholesterol and vascular mortality by age, sex, and blood pressure: a meta-analysis of individual data from 61 prospective studies with 55,000 vascular deaths. *Lancet* 2007;**370**:1829-1839.

10. Porta M. *A dictionary of epidemiology.* Oxford University Press; 2008.

11. Ezatti M, Lopez AD, Rodgers A, Murray CJL, eds. *Comparative quantification of risk. Global and regional burden of disease attributable to selected major risk factors.* World Health Organization; 2004.

12. Roglic G, Unwin N. Mortality attributable to diabetes: estimates for the year 2010. *Diabetes Res Clin Pract* 2010;**87**:15-19.

13. Huxley R, Barzi F, Woodward M. Excess risk of fatal coronary heart disease associated with diabetes in men and women: meta-analysis of 37 prospective cohort studies. *BMJ* 2006;**332**:73-78.

14. Danaei G, Ding EL, Mozaffarian D, Taylor B, Rehm J, Murray CJ, Ezzati M. The preventable causes of death in the United States: comparative risk assessment of dietary, lifestyle, and metabolic risk factors. *PLoS Med* 2009;**6**:e1000058.

15. Tobias M, Taylor R, Yeh LC, Huang K, Mann S, Sharpe N. Did it fall or was it pushed? The contribution of trends in established risk factors to the decline in premature coronary heart disease mortality in New Zealand. *Aust N Z J Public Health* 2008;**32**:117-125.

16. Taylor R, Dobson A, Mirzaei M. Contribution of changes in risk factors to the decline of coronary heart disease mortality in Australia over three decades. *Eur J Cardiovasc Prev Rehabil* 2006;**13**:760-768.

17. Dobson AJ, McElduff P, Heller R, Alexander H, Colley P, D'Este K. Changing patterns of coronary heart disease in the hunter region of New South Wales, Australia. *J Clin Epidemiol* 1999;**52**:761-771.

18. Yusuf S. Two decades of progress in preventing vascular disease. *Lancet* 2002;**360**:2-3.

19. Wald N, Law M. A strategy to reduce cardiovascular disease by more than 80%. 2003;**326**:1419-1426.

20. Mant J, Hicks N. Detecting differences in quality of care: the sensitivity of measures of process and outcome in treating acute myocardial infarction. *BMJ* 1995;**311**:793-796.

21. Pedersen CB. The Danish Civil Registration System. *Scand J Public Health* 2011;**39**:22-25.

22. Weir RA, McMurray JJ, Velazquez EJ. Epidemiology of heart failure and left ventricular systolic dysfunction after acute myocardial infarction: prevalence, clinical characteristics, and prognostic importance. *Am J Cardiol* 2006;**97**:13F-25F.

23. Haastrup B, Gill S, Haghfelt T. Thrombolysis in acute myocardial infarction. The implementation of thrombolytic therapy in a coronary care unit in 1992. *Cardiology* 1994;**85**:397-406.

24. Estess JM, Topol EJ. Fibrinolytic treatment for elderly patients with acute myocardial infarction. *Heart* 2002;**87**:308-311.

25. Randomised trial of intravenous streptokinase, oral aspirin, both, or neither among 17,187 cases of suspected acute myocardial infarction: ISIS-2. ISIS-2 (Second International Study of Infarct Survival) Collaborative Group. *Lancet* 1988;**2**:349-360.

26. Yusuf S, Zucker D, Peduzzi P, Fisher LD, Takaro T, Kennedy JW, Davis K, Killip T, Passamani E, Norris R. Effect of coronary artery bypass graft surgery on survival: overview of 10-year results from randomised trials by the Coronary Artery Bypass Graft Surgery Trialists Collaboration. *Lancet* 1994;**344**:563-570.

27. Keeley EC, Boura JA, Grines CL. Primary angioplasty versus intravenous thrombolytic therapy for acute myocardial infarction: a quantitative review of 23 randomised trials. *Lancet* 2003;**361**:13-20.

28. Freemantle N, Cleland J, Young P, Mason J, Harrison J. beta Blockade after myocardial infarction: systematic review and meta regression analysis. *BMJ* 1999;**318**:1730-1737.

29. Indications for ACE inhibitors in the early treatment of acute myocardial infarction: systematic overview of individual data from 100,000 patients in randomized trials. ACE Inhibitor Myocardial Infarction Collaborative Group. *Circulation* 1998;**97**:2202-2212.

30. Chen ZM, Jiang LX, Chen YP, Xie JX, Pan HC, Peto R, Collins R, Liu LS, COMMIT (ClOpidogrel and Metoprolol in Myocardial Infarction Trial) collaborative group. Addition of clopidogrel to aspirin in 45,852 patients with acute myocardial infarction: randomised placebo-controlled trial. *Lancet* 2005;**366**:1607-1621.

31. Sabatine MS, Cannon CP, Gibson CM, Lopez-Sendon JL, Montalescot G, Theroux P, Claeys MJ, Cools F, Hill KA, Skene AM, McCabe CH, Braunwald E, CLARITY-TIMI 28 Investigators. Addition of clopidogrel to aspirin and fibrinolytic therapy for myocardial infarction with ST-segment elevation. *N Engl J Med* 2005;**352**:1179-1189.

32. Antithrombotic Trialists' Collaboration. Collaborative meta-analysis of randomised trials of antiplatelet therapy for prevention of death, myocardial infarction, and stroke in high risk patients. *BMJ* 2002;**324**:71-86.

33. Oler A, Whooley MA, Oler J, Grady D. Adding heparin to aspirin reduces the incidence of myocardial infarction and death in patients with unstable angina. A meta-analysis. *JAMA* 1996;**276**:811-815.

34. Boersma E, Harrington RA, Moliterno DJ, White H, Theroux P, Van de Werf F, de Torbal A, Armstrong PW, Wallentin LC, Wilcox RG, Simes J, Califf RM, Topol EJ, Simoons ML. Platelet glycoprotein IIb/IIIa inhibitors in acute coronary syndromes: a meta-analysis of all major randomised clinical trials. *Lancet* 2002;**359**:189-198.

35. Fox KA, Poole-Wilson P, Clayton TC, Henderson RA, Shaw TR, Wheatley DJ, Knight R, Pocock SJ. 5-year outcome of an interventional strategy in non-ST-elevation acute coronary syndrome: the British Heart Foundation RITA 3 randomised trial. *Lancet* 2005;**366**:914-920.

36. Yusuf S, Zhao F, Mehta SR, Chrolavicius S, Tognoni G, Fox KK, Clopidogrel in Unstable Angina to Prevent Recurrent Events Trial Investigators. Effects of clopidogrel in addition to aspirin in patients with acute coronary syndromes without ST-segment elevation. *N Engl J Med* 2001;**345**:494-502.

37. Flather MD, Yusuf S, Kober L, Pfeffer M, Hall A, Murray G, Torp-Pedersen C, Ball S, Pogue J, Moye L, Braunwald E. Long-term ACE-inhibitor therapy in patients with heart failure or left-ventricular dysfunction: a systematic overview of data from individual patients. ACE-Inhibitor Myocardial Infarction Collaborative Group. *Lancet* 2000;**355**:1575-1581.

38. Hulten E, Jackson JL, Douglas K, George S, Villines TC. The effect of early, intensive statin therapy on acute coronary syndrome: a meta-analysis of randomized controlled trials. *Arch Intern Med* 2006;**166**:1814-1821.

39. Anand SS, Yusuf S. Oral anticoagulant therapy in patients with coronary artery disease: a meta-analysis. *JAMA* 1999;**282**:2058-2067.

40. Boden WE, O'Rourke RA, Teo KK, Hartigan PM, Maron DJ, Kostuk WJ, Knudtson M, Dada M, Casperson P, Harris CL, Chaitman BR, Shaw L, Gosselin G, Nawaz S, Title LM, Gau G, Blaustein AS, Booth DC, Bates ER, Spertus JA, Berman DS, Mancini GB, Weintraub WS, COURAGE Trial Research Group. Optimal medical therapy with or without PCI for stable coronary disease. *N Engl J Med* 2007;**356**:1503-1516.

41. Wilt TJ, Bloomfield HE, MacDonald R, Nelson D, Rutks I, Ho M, Larsen G, McCall A, Pineros S, Sales A. Effectiveness of statin therapy in adults with coronary heart disease. *Arch Intern Med* 2004;**164**:1427-1436.

42. Al-Mallah MH, Tleyjeh IM, Abdel-Latif AA, Weaver WD. Angiotensin-converting enzyme inhibitors in coronary artery disease and preserved left ventricular systolic function: a systematic review and meta-analysis of randomized controlled trials. *J Am Coll Cardiol* 2006;**47**:1576-1583.

43. Shibata MC, Flather MD, Wang D. Systematic review of the impact of beta blockers on mortality and hospital admissions in heart failure. *Eur J Heart Fail* 2001;**3**:351-357.

44. Pitt B, Zannad F, Remme WJ, Cody R, Castaigne A, Perez A, Palensky J, Wittes J. The effect of spironolactone on morbidity and mortality in patients with severe heart failure. Randomized Aldactone Evaluation Study Investigators. *N Engl J Med* 1999;**341**:709-717.

45. Kjekshus J, Apetrei E, Barrios V, Bohm M, Cleland JG, Cornel JH, Dunselman P, Fonseca C, Goudev A, Grande P, Gullestad L, Hjalmarson A, Hradec J, Janosi A, Kamensky G, Komajda M, Korewicki J, Kuusi T, Mach F, Mareev V, McMurray JJ, Ranjith N, Schaufelberger M, Vanhaecke J, van Veldhuisen DJ, Waagstein F, Wedel H, Wikstrand J, CORONA Group. Rosuvastatin in older patients with systolic heart failure. *N Engl J Med* 2007;**357**:2248-2261.

46. Tavazzi L, Maggioni AP, Marchioli R, Barlera S, Franzosi MG, Latini R, Lucci D, Nicolosi GL, Porcu M, Tognoni G, Gissi-HF Investigators. Effect of rosuvastatin in patients with chronic heart failure (the GISSI-HF trial): a randomised, double-blind, placebo-controlled trial. *Lancet* 2008;**372**:1231-1239.

47. Law M, Wald N, Morris J. Lowering blood pressure to prevent myocardial infarction and stroke: a new preventive strategy. *Health Technol Assess* 2003;**7**:1-94.

48. Pignone M, Phillips C, Mulrow C. Use of lipid lowering drugs for primary prevention of coronary heart disease: meta-analysis of randomised trials. *BMJ* 2000;**321**:983-986.

49. Lewington S, Clarke R, Qizilbash N, Peto R, Collins R, Prospective Studies Collaboration. Age-specific relevance of usual blood pressure to vascular mortality: a meta-analysis of individual data for one million adults in 61 prospective studies. *Lancet* 2002;**360**:1903-1913.

50. Bogers RP, Bemelmans WJ, Hoogenveen RT, Boshuizen HC, Woodward M, Knekt P, van Dam RM, Hu FB, Visscher TL, Menotti A, Thorpe RJ,Jr, Jamrozik K, Calling S, Strand BH, Shipley MJ, BMI-CHD Collaboration Investigators. Association of overweight with increased risk of coronary heart disease partly independent of blood pressure and cholesterol levels: a meta-analysis of 21 cohort studies including more than 300 000 persons. *Arch Intern Med* 2007;**167**:1720-1728.

51. James W, Jackson-Leach R, Mhurchu C, Kalamara E, Shayeghi M, Rigby N, Nishida C, Rodgers A. Overweight and obesity (high body mass index). In: Ezzati M, Lopez AD, Rodgers A and Murray CJL, eds. *Comparative Quantification of Health Risks. Global and Regional Burden of Disease Attributable to Selected Major Risk Factors. Volume 1.* World Health Organization; 2004. p. 497-596.

52. Ezzati M, Henley SJ, Thun MJ, Lopez AD. Role of smoking in global and regional cardiovascular mortality. *Circulation* 2005;**112**:489-497.

53. Bull F, Armstrong TP, Dixon T, Ham S, Neiman A, Pratt M. Physical inactivity. In: Ezzati M, Lopez AD, Rodgers A and Murray CJL, eds. *Comparative quantification of risk. Global and regional burden of disease attributable to selected major risk factors. Volume 1.* World Health Organization; 2004. p. 729-881.

54. Joubert J, Norman R, Lambert EV, Groenewald P, Schneider M, Bull F, Bradshaw D, South African Comparative Risk Assessment Collaborating Group. Estimating the burden of disease attributable to physical inactivity in South Africa in 2000. *S Afr Med J* 2007;**97**:725-731.

55. Hu G, Qiao Q, Tuomilehto J, Balkau B, Borch-Johnsen K, Pyorala K, DECODE Study Group. Prevalence of the metabolic syndrome and its relation to all-cause and cardiovascular mortality in nondiabetic European men and women. *Arch Intern Med* 2004;**164**:1066-1076.

56. Morrish NJ, Wang SL, Stevens LK, Fuller JH, Keen H. Mortality and causes of death in the WHO Multinational Study of Vascular Disease in Diabetes. *Diabetologia* 2001;**44 Suppl 2**:S14-21.

57. Barendregt JJ. The effect size in uncertainty analysis. *Value Health* 2010;**13**:388-391.
